# Right prefrontal activation associated with deviations from expected lipstick texture assessed with functional near-infrared spectroscopy

**DOI:** 10.3389/fnrgo.2024.1331083

**Published:** 2024-05-01

**Authors:** Kazue Hirabayashi, Keith Kawabata Duncan, Keiko Tagai, Yasushi Kyutoku, Ippeita Dan

**Affiliations:** ^1^MIRAI Technology Institute, Shiseido Co., Ltd., Yokohama, Japan; ^2^Applied Cognitive Neuroscience Laboratory, Chuo University, Tokyo, Japan

**Keywords:** functional near-infrared spectroscopy (fNIRS), lipstick, cosmetics, consumer neuroscience, neuromarketing, prefrontal cortex, inferior frontal gyrus (IFG)

## Abstract

**Introduction:**

There is a continuous consumer demand for ever superior cosmetic products. In marketing, various forms of sensory evaluation are used to measure the consumer experience and provide data with which to improve cosmetics. Nonetheless, potential downsides of existing approaches have led to the exploration of the use of neuroimaging methods, such as functional near-infrared spectroscopy (fNIRS), to provide addition information about consumers' experiences with cosmetics. The aim of the present study was to investigate the feasibility of a real-time brain-based product evaluation method which detects the incongruency between a product, in this case lipstick, and a consumer's expectations.

**Method:**

Thirty healthy, female, habitual lipstick users were asked to apply six different lipsticks varying in softness and to rate the softness of and their willingness to pay (WTP) for each lipstick. Cerebral hemodynamic responses in frontal areas were measured with fNIRS during lipstick application and analyzed using the general linear model (GLM). Incongruency scores between softness and expectation were calculated in order to understand how far removed each lipstick was from a participant's optimal softness preference. The correlation between brain activation (beta scores) during the application of each lipstick and the respective incongruency scores from each participant were acquired using semi-partial correlation analysis, controlling for the effects of WTP.

**Results:**

We revealed a significant intra-subject correlation between incongruency scores and activation in the right inferior frontal gyrus (IFG). This confirms that as the texture incongruency scores increased for the lipstick samples, activation in each individual's right IFG also increased.

**Conclusion:**

The correlation observed between incongruency perceived by participants and activation of the right IFG not only suggests that the right IFG may play an important role in detecting incongruity when there is a discrepancy between the perceived texture and the consumer's expectations but also that measuring activity in the IFG may provide a new objective measurement of the consumer experience, thus contributing to the development of superior cosmetics.

## Introduction

Cosmetics companies are constantly trying to develop superior products that meet the needs, preferences, and expectations of consumers. To achieve this, they often conduct extensive sensory evaluations, which were originally used in food and beverage industries (Civille and Carr, 2015), to identify key product attributes that drive consumer satisfaction. Sensory evaluation is now applied not only in the characterization and evaluation of foods and beverages, but also in other industries, including cosmetics, to obtain data upon which informed decisions can be based (Civille and Carr, 2015). Sensory evaluation of cosmetic prototypes, especially luxury cosmetics, is essential to determine the cosmetics' quality and effectiveness.

Sensory evaluation is conventionally done using traditional subjective rating scales administered after the use of the cosmetic. However, this approach may affect the accuracy of evaluations due to biases such as peak-end, in which the highlights, lowlights, and end sensations of an experience bias one's recollections of how that experience felt (Higgins and Altman, 2008; Ares et al., 2013; Scheibehenne and Coppin, 2020). To avoid such biases, real-time sensory evaluation methods for measuring temporal changes in sensations have been developed; they include time-intensity (TI) evaluation (Neilson, 1957; Lee et al., 1986) and TDS (temporal dominance of sensation) (Pineau et al., 2009). As study participants who test cosmetic products are required to be sensitive to relatively small effects of the test product, the act of reporting their own perceptions and sensations may interfere with their natural product experience, potentially producing misleading results (Civille and Carr, 2015).

A promising alternative approach may be to use neuroimaging techniques, which offer a unique opportunity to gain deeper insights into consumers' sensory experiences without interfering with real-life product usage. For instance, functional Near Infrared Spectroscopy (fNIRS) has the potential to evaluate consumers' experiences of cosmetic products during their real-time use (Kawabata Duncan et al., 2019; Hirabayashi et al., 2021). fNIRS is a non-invasive neuroimaging technique that uses near-infrared light to measure hemodynamic signals reflecting changes in hemoglobin concentrations in the brain. It is well-suited for measuring brain activation during real-world tasks and has been used successfully in previous sensory studies such as taste (Okamoto and Dan, 2007; Hu et al., 2014), flavor (Okamoto et al., 2006; Hasegawa et al., 2013), and tactile stimuli (Hong et al., 2017).

Our team has reported that information regarding consumers' monetary evaluations of different cosmetics can be obtained using fNIRS (Kawabata Duncan et al., 2019; Hirabayashi et al., 2021). Our previous studies suggested that the right dorsolateral prefrontal cortex (DLPFC) could be developed as a biomarker for consumer preferences as there was an intra-subject correlation between the right DLPFC activations of participants and their Willingness-To-Pay (WTP) scores during a single, real-time use of cosmetic products. Nonetheless, despite the utility of such an overall evaluation of the cosmetic experience, it is still essential to understand what specific features influence WTP.

One such feature is the texture of cosmetics. In particular, understanding whether or not specific aspects of a product's texture meet or fail to meet consumers' expectations is important in order to optimize product formulations. This process is theoretically described by the schema congruity theory, which posits that the similarity between a product and a broader product category schema affects the processing of incoming sensory information and the resultant evaluation for stimuli such as beverages and scents (Meyers-Levy and Tybout, 1989; Bosmans, 2006; Noseworthy et al., 2014; Lanseng and Sivertsen, 2019). According to this theory, consumers prefer products that align with their existing schemas which are generated based on their memories of previous experiences and which in turn forms their expectations regarding products and brands (Halkias, 2015; Eklund and Helmefalk, 2021). Another study highlighted the importance of understanding consumers' cognitive processes for shaping consumer perceptions and decisions (Mandler, 2014).

In the context of consumer experience of cosmetics such as lipstick, consumers are likely to have formed a schema which incorporates properties such as softness. Excessive deviations from this schema, such as lipsticks which are too soft or too hard, are likely to result in a negative experience and evaluation. While the schema congruity theory provides a robust framework for understanding consumers' evaluations of products, the neural mechanisms underlying these cognitive processes during actual consumer experiences remain unclear. This gap highlights the need for further research to understand how consumers' brains process sensory information in line with or in opposition to their existing schemas, thus influencing their product evaluations.

Thus, the aim of the present study was to investigate the feasibility of a real-time brain-based product evaluation method which detects the incongruency of a product with a consumer's expectations. We focused on lipstick as it is recognized as one of the iconic symbols of luxury cosmetic products (Gerstell et al., 2020) and softness because this is one of the key determinants of the consumer experience with lipstick. To measure the incongruency of six lipsticks which differed in softness, participants evaluated their perception of the softness on a visual analog scale (VAS; from −50 to 50) ranging from *too soft* to *too hard*, resulting in an incongruency score. fNIRS was used to measure the real-time brain activity covering the frontal areas, including the medial Prefrontal Cortex (mPFC), DLPFC, and inferior frontal gyrus (IFG), which we hypothesized, based on previous studies, may be involved in the detection of incongruency (Zhang et al., 2008; van Kesteren et al., 2013; Brod et al., 2015; Yeon et al., 2017; Lin et al., 2018). Due to limitations of the fNIRS system, the anterior cingulate cortex (ACC) was not included. Finally, the relationship between the fNIRS data and incongruency scores was examined using semi-partial intra-subject correlations.

## Methods

### Participants

Thirty healthy, right-handed, Japanese female participants (average age: 29.8 years, SD: 3.7, range: 25–35) who did not have any dermatological conditions resulting in their skin, including the lips, being sensitive or delicate, were recruited via a recruiting company. All participants were native Japanese speakers with no history of neurological, psychiatric, or cardiac disorders. Before the experiment, informed consent was obtained from all participants. They had normal or corrected-to-normal vision and normal color vision. Handedness was assessed by means of the Edinburgh Inventory (Oldfield, 1971). This study was performed in accordance with the principles in the Declaration of Helsinki and approved by the ethical committee at the Shiseido Global Center and Chuo University. As our previous studies only found a relationship between brain activity and subjective ratings in high-frequency cosmetic users (Kawabata Duncan et al., 2019; Hirabayashi et al., 2021), all participants habitually used lipstick at least five times per week and they all preferred to use the common lipstick color called *MAQuillAGE Dramatic Rouge* (Shiseido Co., Ltd., Tokyo, Japan). Their familiarity with lipstick also helped ensure compliance with the experiment instructions. This is a mid-price and popular product which helped facilitate participant recruitment. We conducted this experiment at Chuo University.

### Lipstick samples

Six lipsticks with different levels of softness, including one commercially available at the time of the study and five prototype samples, were prepared (Table 1). The levels of lipstick softness were determined based on the measured physical property values of lipstick products to be balanced in a wide range from too soft to too hard. To avoid the possibility that participants would give a WTP of zero for any given lipstick based solely on their color preferences, the colors of all lipsticks were chosen from an internal color preference guide and fixed for each participant. The physical properties of the lipsticks were measured with a FUDOH Rheometer (Rheotech Co., Ltd., Tokyo, Japan). All lipsticks were presented in the same plain packages.

**Table 1:** Details of lipstick samples.

**Lipstick ID**	**Hardness**	**Softness level**	**Details**
P	5	Very soft	Prototype 1
Q	11		*MAQuillAGE Dramatic Rouge*
R	14		Prototype 2
S	42		Prototype 3
T	95		Prototype 4
U	134	Very hard	Prototype 5

The six different lipsticks used in the experiment were selected based on their measured hardness. Lipstick Q was chosen as the product with the preferred softness based on an internal survey. The remaining lipsticks varied in texture, ranging from too soft to too hard. The color of the lipsticks remained consistent for each variant.

### Experiment design

**Figure 1:**
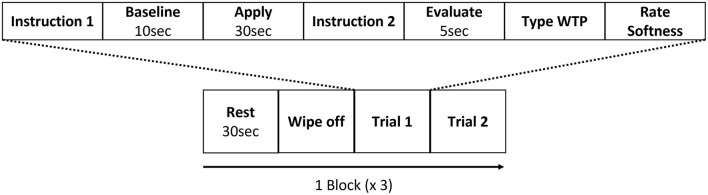
Experimental design (1 block). Each block consisted of two trials. The order of lipsticks and the side to which they were applied were randomized.

For the fNIRS experimental design, one block was composed of 4 periods: Rest, Wipe off, Trial 1, and Trial 2. During the rest period, participants kept still for 30 s (Figure 1). During the wipe off period, they removed the lipstick using a cotton pad with a facial emulsion (*Elixir Superieur lift moist emulsion*, Shiseido Co., Ltd., Tokyo, Japan), which was selected due to its low risk of skin irritation. Participants sat in front of a mirror and a PC monitor that displayed the visual information. A chin rest was used to reduce fNIRS noise associated with motion artifacts. Visual and auditory information was presented using E-Prime (E-Prime 2.0, Psychology Software Tools, Inc., Sharpsburg, PA, USA).

Once the participant finished removing the lipstick, a set of instructions was displayed on the PC monitor informing the participant which lipstick sample (P, Q, R, S, T, or U) they would use next and which side of the lip (left or right) they would apply the lipstick to in this trial. Each lipstick was applied to only one half of the lips to reduce the burden on the delicate skin of the lips. After the participant received the lipstick and was ready to start, 10 s of baseline brain activity was recorded as will be described later. Then, an auditory cue informed the participant that they should begin applying the lipstick and keep applying the lipstick for 30 s until another auditory cue informed them to stop.

Another set of instructions was then displayed on the PC screen which indicated that during the next 5 s, the “Evaluate” period, the participant should think about how much they would like to pay for the lipstick they had just used based on their perception of its softness. Once this 5 s had elapsed, they input their WTP using the keyboard and pressed the Enter key. Next, the VAS appeared on the display and the participant input their perception of the lipstick's softness, from −50 to 50, using a mouse. The scale was presented without the values shown. The left end was labeled “too soft; −50,” the right end was labeled “too hard; 50,” and, hence, the zero point was optimal. After the VAS rating, the participant clicked the button to end the trial. The next trial then began, and the procedure was repeated. Participants repeated this for 3 blocks, and they tried a total of six lipsticks. The absolute value of the rated perceived softness was used as the incongruency score.

### fNIRS data acquisition

**Figure 2:**
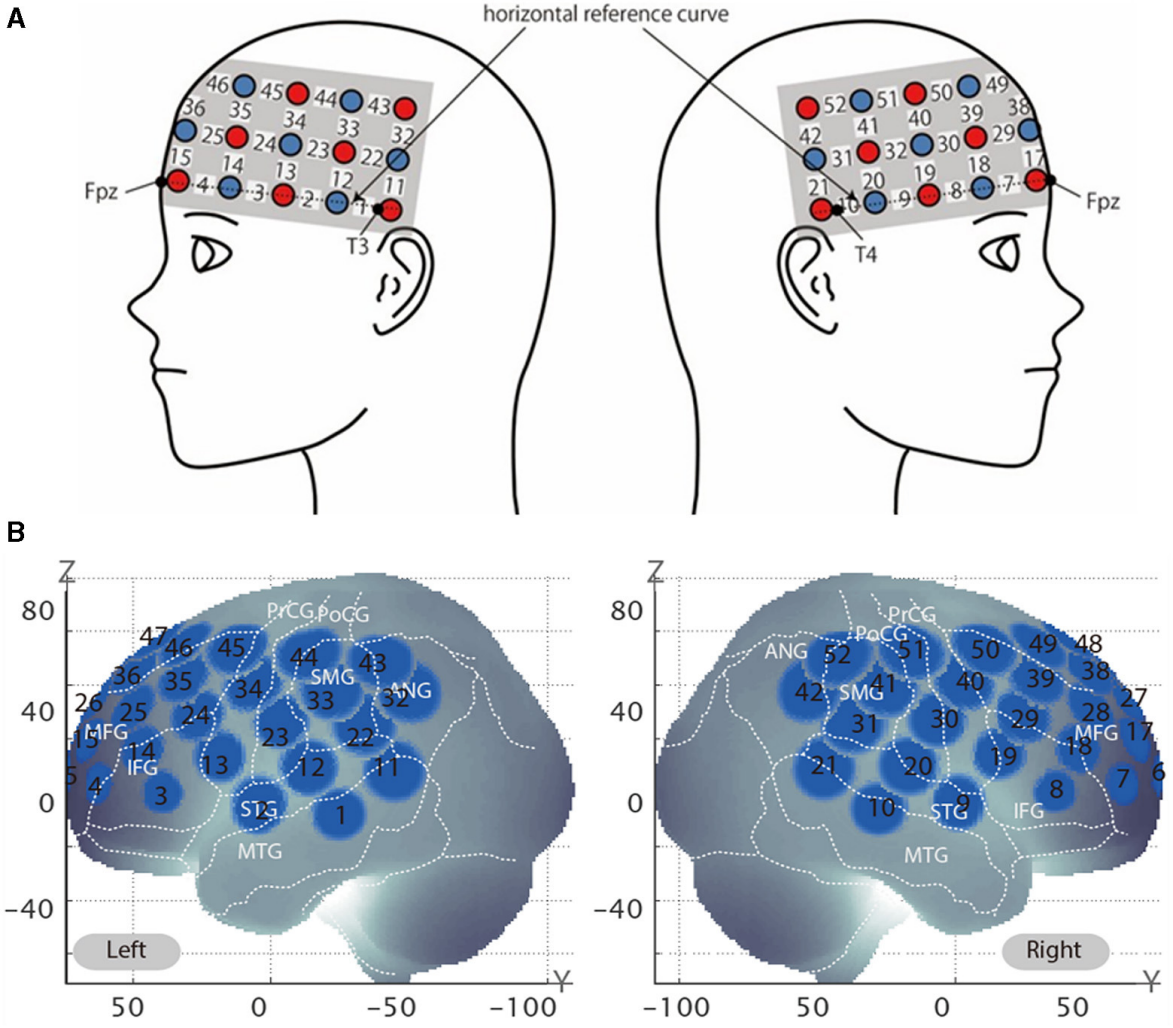
Virtually estimated channel positions. **(A)** Spatial profiles of functional near-infrared spectroscopy (fNIRS) channels. This figure shows left- and right-side views of the probe arrangements with fNIRS channel orientation. Detectors are indicated with blue circles, illuminators with red circles, and channels with white squares. **(B)** The estimated channel locations on the brain for both left- and right-side views are shown. Corresponding channel numbers are shown in black. The circles indicate the spatial variability associated with the estimation exhibited in Montreal Neurological Institute (MNI) space.

In the experiment, hemodynamic responses of the brain were measured with a multichannel fNIRS optical topography system ETG-4000 (Hitachi Medical Corporation, Kashiwa, Japan) using dual wavelengths of near-infrared light (695 and 830 nm) at a 10 Hz sampling rate. The optical data was analyzed based on the modified Beer-Lambert Law (Cope et al., 1988). Accordingly, signals reflecting concentration changes of the oxygenated hemoglobin (oxy-Hb) and deoxygenated hemoglobin (deoxy-Hb) were obtained in units of millimolar · millimeter (mM · mm) (Maki et al., 1995). For our analysis, we focused on the oxy-Hb signal because it has been shown to be more reliable than the deoxy-Hb signal (Dravida et al., 2018) and only the oxy-Hb signal showed a correlation with behavior in our previous research (Kawabata Duncan et al., 2019; Hirabayashi et al., 2021). We used a 3 × 11 multichannel probe holder consisting of 17 illuminating and 16 detecting probes arranged alternately at an inter-probe distance of 3 cm, producing a total of 52 channel positions for measurement (Figure 2A). The probe was mounted using the international 10–20 system as a reference point. First, the multichannel probe holder was placed such that the detector in the middle of the lowest row corresponded to the Fpz. Then, the illuminators and detectors in the lowest row were matched to the horizontal reference curve, which was determined by a straight line connecting T3-Fpz-T4 (Klem et al., 1999; Jurcak et al., 2007).

### Registration of fNIRS channels to MNI space

After fNIRS measurements, the location of all the optodes and landmarks, such as the nasion, inion, CZ, and bilateral preauricular reference points, were acquired using the Polhemus Patriot digitizer (Polhemus, Colchester, VT, USA). We employed probabilistic registration to register fNIRS data to MNI (Montreal Neurological Institute) standard brain space (Tsuzuki et al., 2007; Tsuzuki and Dan, 2014). The spatial registration data were registered with macro-anatomical labeling (Okamoto et al., 2004; Okamoto and Dan, 2005) in reference to Brodmann's atlas (BA) (Rorden and Brett, 2000) and secondarily to the macro-anatomical labeling in LPBA40 (Shattuck et al., 2008). Data for three participants' data were removed because of positional errors in probe placement.

### fNIRS data analysis

For the first-level analysis, oxy-Hb time series data were analyzed with in-house MATLAB analysis tools developed in the Applied Cognitive Neuroscience Laboratory at Chuo University (available upon request). First, we used Platform for Optical Topography Analysis Tools (POTATo) (Sutoko et al., 2016) for data preprocessing. The individual oxy-Hb time series data of each of the 52 channels were preprocessed with a first-degree polynomial fitting and high-pass filter using cut-off frequencies of 0.01 Hz to remove baseline drift and a 0.8 Hz low-pass filter to remove heartbeat and pulse noise. Channels with signal fluctuations of 10% or less were considered to have poor measurement quality and were excluded from the analysis. Then, wavelet minimum description length (Wavelet-MDL) was applied to remove the effects of measurement noise, such as respiration and heart motion, from the remaining channels (Jang et al., 2009). After preprocessing, we conducted general linear model (GLM) analysis with regression of HRF on the oxy-Hb time series data from each channel for each subject. Basis functions used for GLM analysis were generated from the HRFs ***h***(**τ**_***p***_, ***t***) (Equation 1; Friston et al., 1998).


(1) $$h\left(\tau_{p},\;t\right)=\frac{t^{\tau_{p}}e^{-t}}{\left(\tau_{p}\right)!}-\frac{t^{\tau_{p}+\tau_{d}}e^{-t}}{A\left(\tau_{p}+\tau_{d}\right)!}$$

Here, *t* represents a point in the time series, **τ**_**p**_represents the first peak delay, set as 6 s, and **τ**_**d**_ represents the second peak delay, set as 16 s. *A*, which is the amplitude ratio between the first and second peaks, was set to 6 s as is commonly done in typical fMRI studies. The first and second derivatives were included to further eliminate the influence of noise of individual data.

Basis functions ***f***(**τ**_***p***_, ***t***) were generated by convolving the HRF ***h***(**τ**_***p***_, ***t***) with a boxcar function ***u***(***t***) (Equation 2),


(2) $$f\left(\tau_{p},\;t\right)=h\left(\tau_{p},\;t\right)⊗u\left(t\right)$$

where the symbol ⊗ denotes convolution integral. The basic functions were used to compose each regressor described as follows. The regressors included in the GLM analysis were 30 s Apply, 5 s Evaluate, Type WTP, and Rate Softness (VAS) for each trial. The β value is used as an estimate of the HRF prediction of the oxy-Hb signal. A total of six β values corresponding to lipstick samples P, Q, R, S, T, and U were calculated for both the Apply period, as β_*AP*_, β_*AQ*_, β_*AR*_, β_*AS*_, β_*AT*_, and β__*AU*__, and the Evaluate period, as β_*EP*_, β_*EQ*_, β_*ER*_, β_*ES*_, β_*ET*_, and β_*EU*_. Single β values were obtained for the Type WTP and Rate Softness (VAS) periods combined as β_*type*_. Consequently, a total of 13 β values were obtained. Figure 6 shows the observed hemodynamic responses and the predicted responses, which were determined by combining regressors adjusted for each participant with personalized adaptive GLMs.

Next, we calculated the Spearman correlation coefficient for each of the 52 channels of each participant between the incongruency scores and the apply period of β values from β_*AP*_ to β_*AU*_ in order to investigate whether there was a significant intrasubject correlation. The inclusion of the incongruency scores differs compared to our previous experimental designs (Kawabata Duncan et al., 2019; Hirabayashi et al., 2021). In this study, we specifically aimed to investigate the intra-subject correlation between the incongruency scores for products P to U, and β_*AP*_ to β_*AU*_. However, we also needed to consider the presence of a third variable, WTP, in order to ensure that the correlations were explained as the association between two random variables after eliminating the effect of the other random variables (Kim, 2015). Therefore, we utilized semi-partial correlation (Tabachnick and Fidell, 2014), a type of statistical analysis which can take into account the effect of a third variable on the correlation between two other variables. To apply this method to our experiment, first the Spearman correlation coefficient within subject between the incongruency score of softness (*Incongruency score*) and β values during application period *(*β_*A*_*)* for the six lipsticks was calculated as ρ_*Incongruency score*._β__*A*__. That between WTP scores and β values during the application period for the six lipsticks was given as ρ_*WTP*._β__*A*__. That between the WTP scores and the incongruency scores was given as ρ_*WTP*.*Incongruency score*_. From these three Spearman correlation coefficients, the semi-partial correlation between the incongruency scores and beta values during the application period controlling the effects of WTP values, ρ_*Incongruency score*._β__*A*_|*WTP*_, was calculated as below (Equation 3).


(3) $$\begin{array}{l} \;\rho_{Incongruency\;score.\beta_{A}|WTP}=\\ \frac{\rho_{Incongruency\;score.\beta_{A}\;}-\rho_{WTP.\beta_{A}\;}\;*\;\rho_{WTP.Incongruency\;score}{\;}_{\;}}{\sqrt{1-\rho^{2}{\;}_{WTP.Incongruency\;score}}} \end{array}$$

Intra-personal correlation analyses were performed as in our previous studies (Kawabata Duncan et al., 2019; Hirabayashi et al., 2021), therefore once the ρ_*Incongruency score*._β__*A*_|*WTP*_ was calculated for each subject, the value of each channel was converted into a Z score using Fisher's *r*-to-*z* transformation. Prior to conducting statistical analysis, it was essential to consider a reasonable effect size for the dataset of 27 participants. A power analysis was performed using G^*^Power 3.1.9.7 (Faul et al., 2007), with the following parameters: a sample size of 27, a one sample *t*-test for each channel, a type-I threshold (α) of 0.05, and power (1- β) of >0.8. This analysis yielded an effect size (Cohen's *d*) of 0.56, which was deemed reasonable (medium > 0.5) (Cohen, 2013). Subsequently, a one sample *t*-test was conducted to determine whether the average Z scores differed from 0. Following this, Fisher's *z*-to-*r* inverse transformation was applied to convert the average Z of all participants to the average coefficients of all participants.

## Results

### Behavioral performance: incongruency scores for softness

**Figure 3:**
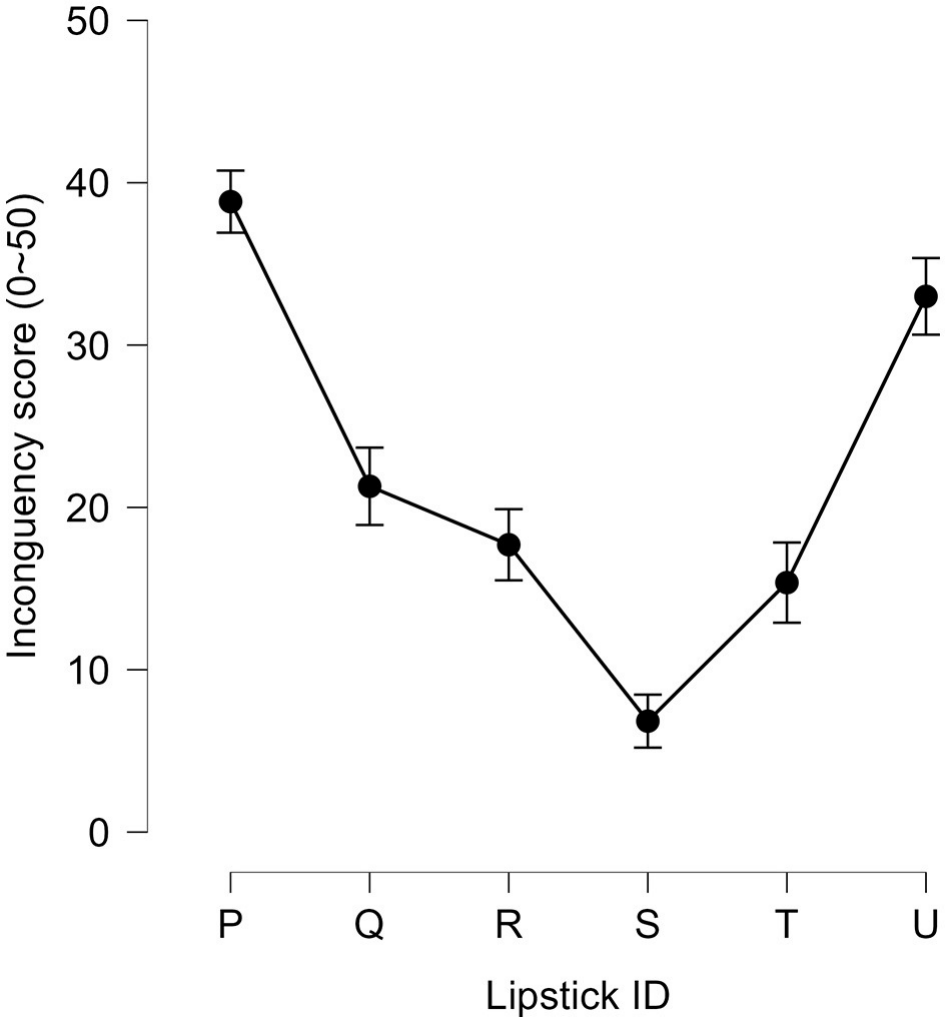
Average absolute values of incongruency scores (0-50) for six lipsticks. The graph shows the group average incongruency scores calculated by taking the absolute values of the perceived softness scores. P, Q, R, S, T, and U represent the six different lipsticks on a softness texture scale (soft to hard). Error bars represent the standard error of the mean.

The incongruency scores for the six lipsticks are shown in Figure 3. During the test, participants rated their perception of the softness of the lipsticks from too hard (−50) to too soft (50) using a VAS. The values were converted into an incongruency score by taking the absolute value to visualize the distance of each VAS rating from 0, to ultimately produce the optimal softness score. In other words, if participants felt that the lipstick was of optimal softness, their incongruency score would be closer to 0. These incongruency scores were analyzed with a one-way repeated-measures ANOVA with lipstick samples as within-subject factors using JASP (Version 0.17.1). This revealed a significant main effect of lipstick [*F*_(5, 145)_ = 29.4, *p* < 0.001, η^2^ = 0.50, also see Table 2], suggesting that participants were able to discern differences in softness among the six different lipsticks. The incongruency scores, which represent deviation from the optimal softness score of 0, varied significantly across the lipstick samples. A lower incongruency score indicates that participants perceived the lipstick to be closer to the optimal softness.

**Table 2:** Summary of the behavioral performance results.

**Lipstick ID**	**N**	**WTP score (JPY)**		**Incongruency score**	
		**Mean**	**SEM**	**Mean**	**SEM**
P	30	953.3	181.7	38.8	2.2
Q	30	1,641.0	213.8	21.3	2.5
R	30	1,865.0	202.2	17.7	2.3
S	30	2,032.7	202.5	6.8	1.4
T	30	1,600.0	166.5	15.4	2.4
U	30	979.7	154.0	33.0	2.6

Mean WTP and incongruency scores for all six lipsticks (*n* = 30). SEM, Standard Error of the Mean.

### Behavioral performance: WTP

**Figure 4:**
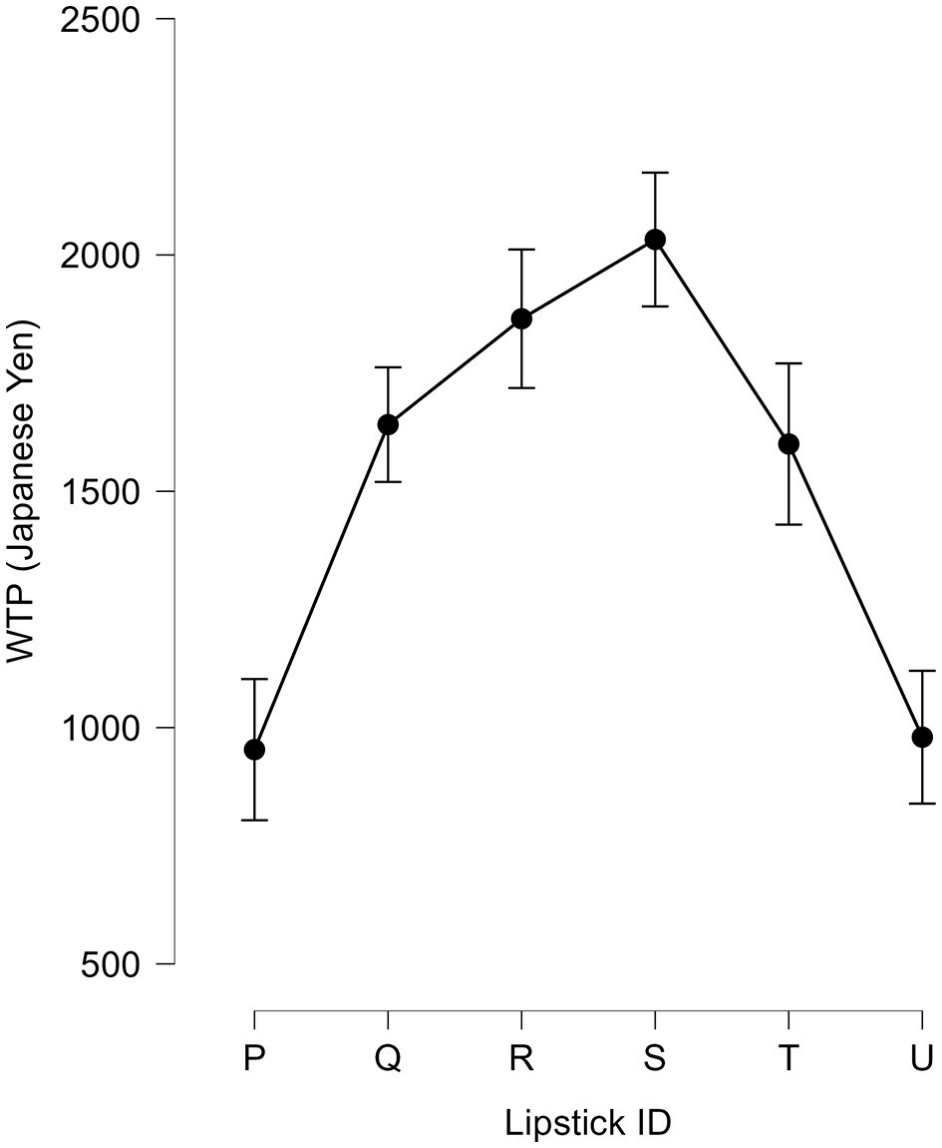
Average WTP (willingness-to-pay) for six lipsticks. The graph shows the group average WTP scores for six different lipsticks in Japanese yen (JPY). Error bars represent the standard error of the mean.

In addition to the perceived softness scores, participants were asked to rate their WTP for each lipstick. The average WTPs for the six lipsticks varied significantly, as shown in Figure 4. These data were analyzed using a one-way repeated measures ANOVA with lipstick samples as within-subject factors. This revealed a significant main effect of lipstick [*F*_(5, 145)_ = 9.57, *p* < 0.001, η^2^ = 0.25, also see Table 2], thus suggesting that the perceived softness significantly influences the overall evaluation of a lipstick. In other words, the greater the incongruency, the lower the participant's willingness to pay.

### Intra-personal correlation

**Figure 5:**
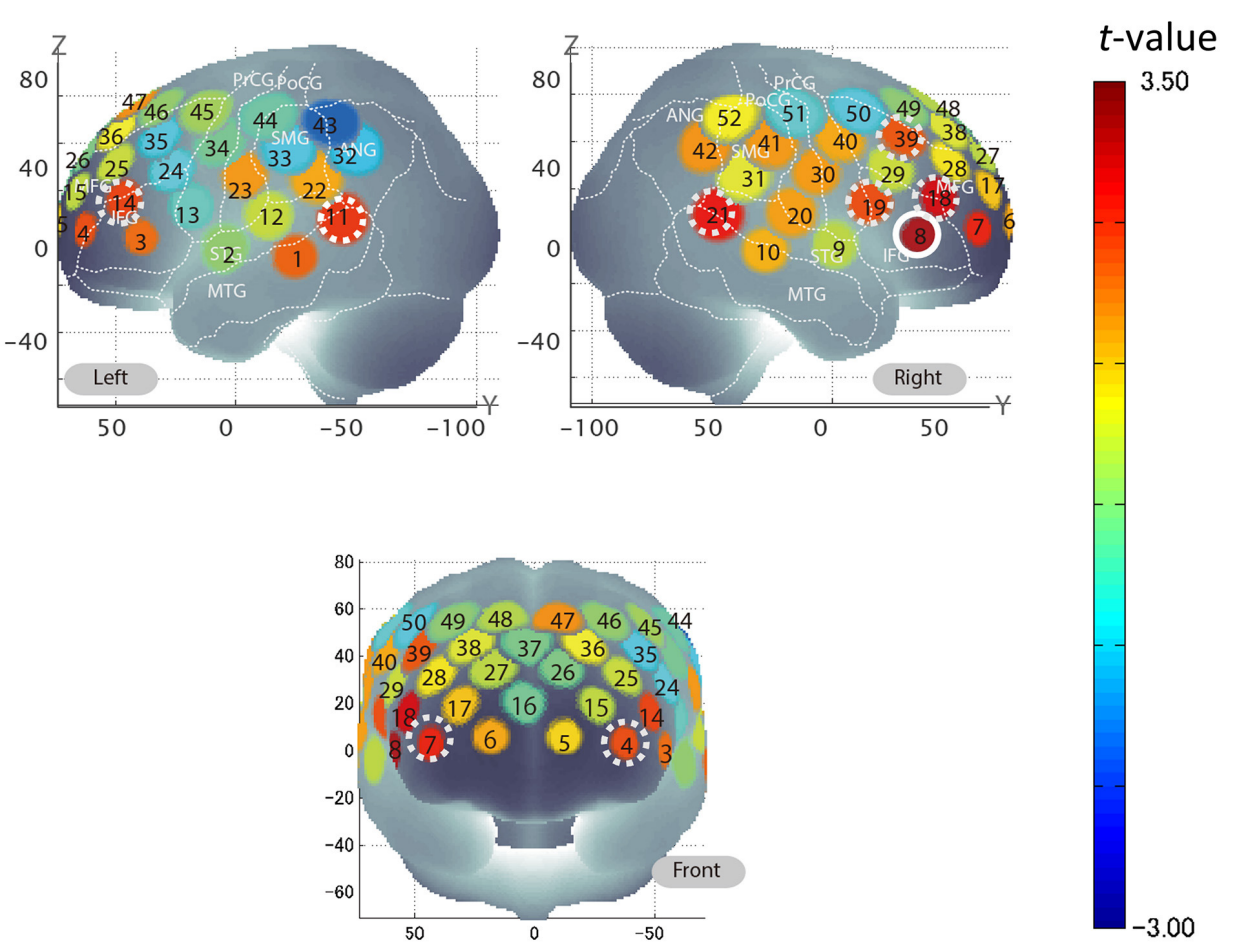
Group average of semi-partial correlation coefficients across 52 channels for incongruency score. Based on the *t*-values resulting from one-sample *t*-tests for the mean *Z* of **ρ**_**Incongruency** **score.**_**β**__**A**_**|softness**_ for each channel, the corresponding values were plotted according to the color-bar. Ch 8, covering the right IFG, is encircled by a solid white line as it was found to be significantly different from 0 and demonstrated a sufficient effect size. Additionally, channels that were found to be statistically significant (Ch 4, 7, 11, 14, 18, 19, 21, and 39) are encircled by dotted white lines.

**Figure 6:**
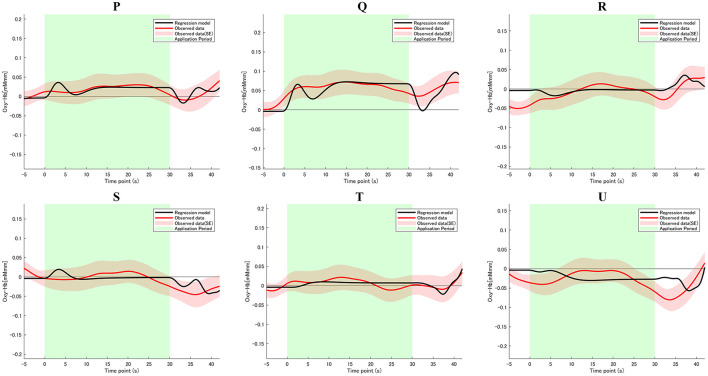
fNIRS time series data for Ch 8 during lipstick application. The graphs show the observed and predicted time-series data for fNIRS from all channels for six lipstick samples (P, Q, R, S, T, and U), averaged across all subjects, *n* = 27. The red lines indicate the observed oxygenated hemoglobin (oxy-Hb) signal, and the black lines indicate predicted hemodynamic responses. Standard errors are shown as pale red (*n* = 27). The green highlighted area is the 30 s of the application period.

We investigated whether the brain activation in any specific brain area reflected the softness incongruency of the lipsticks. We first examined the correlation coefficients of all channels of all subjects, then computed the value of ρ_*incongruency score*._β__*Apply*_|*WTP*_ and converted these coefficients into Z values (see Supplementary material for the data). The results of the mean *Z* in the group significantly differed from 0 in multiple channels (Table 3). The results for all 52 channels are displayed in Figure 5, and detailed numerical values can be found in the Supplementary material. The one-sample *t*-test results for this channel were significant [*t*_(26)_ = 3.15; *p* < 0.01; *d* = 0.61; see Supplementary material] and, based on the power analysis, only channel 8 showed an effect size *(d)* exceeding the reasonable threshold of 0.56. This was derived from a mean Z score of 0.31 with a standard error of the mean of 0.51. Figure 6 shows the observed hemodynamic response and the predicted response, which was created by combining regressors adjusted for each participant with the personalized adaptive GLM. Ch 8 covered the right IFG, based on the results of the MNI coordinates (x = 58.0, y = 36.3, z = 0.7, SD = 8.3), with microanatomical estimation of the right IFG via LBPA40 (Shattuck et al., 2008). After *z* to *r* conversion, the group average of mean ρ_*Incongruency score*._β__*Apply*_|*softness*_ was 0.30 at Ch 8.

**Table 3:** Channels of mean Z in the group significantly differed from 0.

**Channel**	**Mean R**	**Mean Z**	**SD**	***t***	***p***	***d***	**1-β**	***df***
4	0.29	0.30	0.70	2.19	0.04	0.42	0.56	26
7	0.23	0.24	0.50	2.46	0.02	0.47	0.66	26
8	0.30	0.31	0.51	3.15	0.004	0.61	0.86	26
11	0.23	0.24	0.52	2.36	0.03	0.45	0.62	26
14	0.30	0.31	0.71	2.27	0.03	0.44	0.59	26
18	0.32	0.33	0.59	2.88	0.01	0.55	0.79	26
19	0.27	0.28	0.65	2.18	0.04	0.43	0.55	25
21	0.20	0.20	0.40	2.54	0.02	0.5	0.69	25
39	0.19	0.19	0.47	2.12	0.04	0.41	0.53	26

Channels for which the mean Z-score, derived from ρ_*Incongruency score*._β__*Apply*_|*WTP*_, was significantly different from 0 in the one-sample *t*-test. SD, standard deviation; *t, t*-value; *d*, Cohen's *d; p, p*-value; *1-*β, power; *n* = 27. Additionally, the Mean R, calculated from the Mean Z using the inverse Fisher's *z*-to-*r* transformation, is also provided.

## Discussion

The aim of the present study was to investigate the feasibility of a real-time brain-based product evaluation method which detects the incongruency between a product and a consumer's expectations. We looked for brain areas where activation was related to the incongruency of the softness of lipsticks. We found significant intra-subject correlations between the incongruency score and activation in the right IFG. These correlations were revealed using semi-partial correlation analysis, which allowed us to control the influence of the third variable of WTP. This is the first step toward a commercially applicable brain-based measurement which can help reveal specific features of a product, such as the softness of a lipstick, which are failing to meet consumer expectations and, therefore, assist in product development.

Importantly, the current study builds upon our prior research by confirming intra-subject correlations multiple times (Kawabata Duncan et al., 2019; Hirabayashi et al., 2021). By focusing on the incongruity of a cosmetic item's texture with participants' expectations, we have expanded our understanding of the relationship between the monetary evaluation of cosmetic products and the cortical activation concurrently evoked. Unlike previous studies that solely relied on WTP values to assess overall scores of cosmetics, our investigation specifically examined how texture features influence WTP scores. These findings offer valuable insights that can inform product formulation and optimize texture. Furthermore, our results feature the significance of individual variations in brain activity, emphasizing the necessity of intra-subject analyses to capture meaningful associations.

Moreover, applying schema congruency theory to lipsticks leads to the prediction that the more the softness of a lipstick deviates from a consumer's optimal preference, the worse the consumer evaluation will be (Meyers-Levy and Tybout, 1989; Huang et al., 2013). Consistent with this, we found an inverse correlation between softness incongruency and WTP, which also confirms the critical role of softness in the experience of lipstick. Crucially, we observed an intra-subject correlation between the incongruency score of the lipsticks and activation in the right IFG. We suggest that the greater activation in the right IFG can be interpreted as the brain's response to the incongruency between a product and a consumer's expectations. This is consistent with previous studies such as that of Sherman et al. (2016), who found that the right IFG is sensitive to the discrepancy between expectation and decision and that of Allen et al. (2016), who observed tactile mismatch responses in the right IFG using fMRI.

In addition to the activation of the right IFG, we observed that several other cortical regions were associated with the incongruency score. This might be attributable to the involvement of working memory. In our study, participants engaged in the evaluation of the physical properties of a lipstick, and this is in line with the concept of exploratory decision-making (Daw et al., 2006; Krug et al., 2014; Domenech and Koechlin, 2015). Once the lipstick incongruity score is formed individually, the respective WTP values should be determined. Previously, neuroimaging of working memory during sensory evaluation was performed with fNIRS. Okamoto et al. (2011) found that the right hemispheric lateral PFC, including the DLPFC and extending to parts of the ventrolateral prefrontal cortex (VLPFC) such as the IFG, was activated during memory retrieval. Considering these findings, we postulated that the prefrontal cortices are important for integrating multiple sensory cues including tactile and taste information. Thus, our study provides a crucial key to understanding cognitive processes that occur during tactile sensory evaluations, which constitutes an important step in the development and formulation of cosmetic products.

Due to the limitations of fNIRS, specifically that it is unable to detect signals from structures that do not lie directly below the scalp, we are not able to measure activation in areas, such as the ACC, which have been associated with the processing of incongruity (Noseworthy et al., 2014; Kolling et al., 2016; Clairis and Lopez-Persem, 2023). Therefore, it may be the case that such areas would provide more robust biomarkers for incongruency during product experience. However, measuring these areas with fMRI is at odds with the main objective of this research, which is to measure consumers' real-time experience of cosmetics in as natural a setting as possible; the application of cosmetics in an fMRI scanner is very difficult.

## Conclusion

In conclusion, this study represents the first step in the development of a commercially applicable brain-based method of detecting the degree of incongruency a consumer experiences during their real-time use of a product. In this case, our target was the incongruency experienced due to varying softnesses of lipsticks; however, future work could investigate the applicability of this method to other product features and other products. In addition, in the current study the brain signals were averaged based on time domain, which may have led to the omission of useful information regarding the temporal evolution of incongruency.

Future studies could investigate the possibility of a different approach which does not use temporal averaging, and this could be combined with temporal sensory evaluation methods such as TDS to determine what sensations are driving the incongruency more generally. Such methods could potentially be expanded to areas such as product development and marketing for other cosmetic products' textures, scents, and packaging evaluation. They could provide neurobiologically based understanding for complex consumer preferences for various products.

Overall, further research with fNIRS holds significant potential for advancing our understanding of consumers' cosmetics experiences. This knowledge can be applied to product development and marketing strategies, leading to the creation of more appealing and successful products.

## Data availability statement

The raw data supporting the conclusions of this article will be made available by the authors, without undue reservation.

## Ethics statement

The studies involving humans were approved by the Institutional Ethics Committee of Chuo University. The studies were conducted in accordance with the local legislation and institutional requirements. The participants provided their written informed consent to participate in this study.

## Author contributions

KH: Conceptualization, Investigation, Methodology, Formal analysis, Writing – original draft. KK: Conceptualization, Methodology, Funding acquisition, Supervision, Writing – review & editing. KT: Supervision, Writing – review & editing, Resources. YK: Writing – review & editing, Data curation, Methodology, Validation. ID: Methodology, Writing – review & editing, Conceptualization, Funding acquisition, Investigation, Supervision.
