# Supplementary material for: Right prefrontal activation associated with deviations from expected lipstick texture assessed with functional near-infrared spectroscopy
Source: Front Neuroergon. 2024 May 1;5:1331083. doi: 10.3389/fnrgo.2024.1331083 (PMC11094294; doi:10.3389/fnrgo.2024.1331083)
Supplement: Supplementary file 1 [file Data_Sheet_1.DOCX]

Supplementary Material

**Table S1 Spearmen correlation coefficients, semi-partial correlation coefficients, and Z scores**

|  | Sub1 | Sub1 | Sub1 | Sub2 | Sub2 | Sub2 | Sub3 | Sub3 | Sub3 | Sub4 |  | Sub4 | Sub4 |
| --- | --- | --- | --- | --- | --- | --- | --- | --- | --- | --- | --- | --- | --- |
| Channel | *r* | *sr* | *Z* | *r* | *sr* | *Z* | *r* | *sr* | *Z* | *r* |  | *sr* | *Z* |
| 1 | 0.60 | 0.24 | 0.24 | 0.94 | 0.88 | 1.36 | -0.33 | -0.40 | -0.43 | -0.60 |  | -0.26 | -0.27 |
| 2 | 0.49 | 0.15 | 0.15 | 0.20 | 0.06 | 0.06 | -0.70 | -0.60 | -0.70 | -0.77 |  | -0.67 | -0.82 |
| 3 | 0.71 | 0.54 | 0.61 | -0.09 | -0.17 | -0.17 | 0.03 | -0.20 | -0.21 | -0.66 |  | -0.43 | -0.46 |
| 4 | 0.37 | -0.01 | -0.01 | 0.94 | 0.98 | 2.39 | 0.33 | -0.02 | -0.02 | 0.14 |  | 0.08 | 0.08 |
| 5 | 0.09 | 0.16 | 0.16 | 0.49 | 0.37 | 0.39 | 0.21 | 0.26 | 0.26 | 0.43 |  | 0.30 | 0.30 |
| 6 | 0.03 | -0.64 | -0.76 | 0.94 | 0.92 | 1.61 | 0.33 | 0.46 | 0.50 | 0.43 |  | 0.36 | 0.38 |
| 7 | 0.83 | 0.13 | 0.13 | 0.89 | 0.85 | 1.25 | -0.03 | 0.08 | 0.08 | 0.14 |  | -0.20 | -0.20 |
| 8 | -0.14 | -0.53 | -0.59 | 0.77 | 0.61 | 0.71 | -0.39 | -0.42 | -0.44 | -0.09 |  | 0.10 | 0.10 |
| 9 | -0.60 | -0.89 | -1.41 | 0.14 | 0.08 | 0.08 | -0.52 | -0.62 | -0.73 | -0.14 |  | 0.16 | 0.17 |
| 10 | -0.14 | -0.89 | -1.41 | -0.20 | -0.28 | -0.28 | -0.21 | -0.08 | -0.08 | 0.14 |  | 0.36 | 0.38 |
| 11 | 0.31 | 0.05 | 0.05 | 0.83 | 0.74 | 0.96 | -0.33 | -0.28 | -0.29 | -0.26 |  | -0.41 | -0.44 |
| 12 | 0.71 | 0.04 | 0.04 | 0.60 | 0.55 | 0.62 | -0.21 | -0.20 | -0.20 | -0.54 |  | -0.31 | -0.32 |
| 13 | 0.37 | 0.49 | 0.54 | -0.43 | -0.53 | -0.59 | 0.03 | -0.08 | -0.08 | -0.71 |  | -0.51 | -0.56 |
| 14 | -0.09 | 0.49 | 0.54 | 0.49 | 0.57 | 0.65 | 0.33 | 0.22 | 0.23 | 0.89 |  | 0.92 | 1.59 |
| 15 | 0.14 | -0.34 | -0.35 | 0.26 | 0.27 | 0.28 | 0.09 | -0.07 | -0.07 | 0.66 |  | 0.56 | 0.63 |
| 16 | 0.43 | 0.57 | 0.65 | -0.31 | -0.35 | -0.36 | 0.15 | 0.24 | 0.25 | 0.37 |  | 0.41 | 0.44 |
| 17 | 0.43 | 0.57 | 0.65 | 0.89 | 0.76 | 0.99 | -0.09 | -0.05 | -0.05 | 0.43 |  | 0.20 | 0.20 |
| 18 | 0.60 | 0.89 | 1.41 | 0.71 | 0.69 | 0.84 | 0.21 | -0.28 | -0.29 | -0.03 |  | 0.05 | 0.05 |
| 19 | -0.66 | -0.54 | -0.60 | 0.77 | 0.88 | 1.38 | -0.52 | -0.50 | -0.55 | -0.26 |  | 0.07 | 0.07 |
| 20 | 0.26 | -0.68 | -0.83 | 0.71 | 0.69 | 0.84 | 0.03 | 0.10 | 0.10 | -0.26 |  | 0.07 | 0.07 |
| 21 | 0.37 | 0.06 | 0.06 | 0.14 | 0.03 | 0.03 | -0.27 | 0.09 | 0.09 | -0.26 |  | 0.07 | 0.07 |
| 22 | -0.20 | 0.33 | 0.34 | 0.49 | 0.46 | 0.50 | 0.21 | 0.14 | 0.14 | 0.26 |  | 0.41 | 0.44 |
| 23 | -0.31 | -0.12 | -0.12 | 0.66 | 0.58 | 0.66 | 0.58 | 0.16 | 0.16 | 0.71 |  | 0.80 | 1.11 |
| 24 | -0.49 | -0.15 | -0.15 | 0.09 | 0.17 | 0.17 | 0.33 | -0.02 | -0.02 | -0.54 |  | -0.18 | -0.18 |
| 25 | -0.20 | 0.33 | 0.34 | 0.37 | 0.26 | 0.26 | 0.33 | -0.02 | -0.02 | 0.14 |  | 0.08 | 0.08 |
| 26 | 0.03 | 0.08 | 0.08 | 0.77 | 0.70 | 0.87 | -0.64 | -0.71 | -0.89 | 0.31 |  | 0.39 | 0.42 |
| 27 | -0.26 | 0.54 | 0.60 | 0.54 | 0.57 | 0.65 | -0.52 | -0.50 | -0.55 | -0.09 |  | 0.34 | 0.36 |
| 28 | 0.14 | 0.53 | 0.59 | 0.94 | 0.88 | 1.36 | 0.03 | -0.20 | -0.21 | -0.14 |  | 0.13 | 0.13 |
| 29 | -0.14 | 0.34 | 0.35 | 0.49 | 0.37 | 0.39 | 0.21 | -0.16 | -0.17 | 0.20 |  | 0.10 | 0.10 |
| 30 | 0.26 | -0.18 | -0.18 | 0.49 | 0.40 | 0.43 | -0.21 | -0.14 | -0.14 | 0.49 |  | 0.30 | 0.30 |
| 31 |  |  |  | 0.49 | 0.46 | 0.50 | 0.58 | 0.58 | 0.66 | -0.60 |  | -0.26 | -0.27 |
| 32 | 0.14 | 0.45 | 0.49 | -0.26 | -0.40 | -0.42 | -0.39 | -0.36 | -0.37 | -0.89 |  | -0.56 | -0.63 |
| 33 | -0.60 | -0.02 | -0.02 | -0.77 | -0.88 | -1.38 | 0.64 | 0.35 | 0.37 | -0.20 |  | -0.11 | -0.12 |
| 34 | -0.03 | -0.65 | -0.78 | -0.26 | -0.30 | -0.31 | 0.21 | -0.16 | -0.17 | -0.09 |  | 0.13 | 0.13 |
| 35 | -0.14 | 0.63 | 0.73 | -0.49 | -0.60 | -0.70 | 0.46 | 0.25 | 0.26 | 0.14 |  | -0.05 | -0.05 |
| 36 | -0.09 | 0.49 | 0.54 | 0.37 | 0.26 | 0.26 | 0.33 | 0.58 | 0.67 | -0.37 |  | -0.13 | -0.13 |
| 37 | 0.03 | 0.00 | 0.00 | 0.77 | 0.67 | 0.81 | -0.70 | -0.60 | -0.70 | -0.09 |  | 0.08 | 0.08 |
| 38 | 0.14 | 0.45 | 0.49 | 0.77 | 0.67 | 0.81 | 0.33 | 0.22 | 0.23 | -0.37 |  | 0.00 | 0.00 |
| 39 | -0.49 | 0.14 | 0.14 | 0.77 | 0.81 | 1.12 | 0.33 | 0.40 | 0.43 | -0.26 |  | -0.03 | -0.03 |
| 40 | -0.49 | 0.14 | 0.14 | 0.49 | 0.36 | 0.37 | 0.33 | 0.40 | 0.43 | 0.31 |  | 0.39 | 0.42 |
| 41 | 0.71 | 0.33 | 0.34 | 0.94 | 0.94 | 1.72 | 0.33 | 0.34 | 0.36 | -0.03 |  | 0.10 | 0.10 |
| 42 | -0.14 | 0.48 | 0.52 | 0.94 | 0.94 | 1.72 | 0.09 | 0.41 | 0.44 | -0.54 |  | -0.18 | -0.18 |
| 43 | 0.14 | 0.45 | 0.49 | -0.49 | -0.60 | -0.70 | -0.27 | -0.27 | -0.28 | -0.54 |  | -0.61 | -0.70 |
| 44 | 0.77 | 0.48 | 0.52 | -0.60 | -0.75 | -0.97 | -0.03 | -0.34 | -0.35 | -0.03 |  | 0.15 | 0.15 |
| 45 | 0.71 | 0.62 | 0.72 | -0.49 | -0.60 | -0.70 | 0.33 | -0.02 | -0.02 | -0.20 |  | -0.10 | -0.10 |
| 46 | 0.89 | 0.36 | 0.37 | 0.71 | 0.72 | 0.90 | 0.33 | 0.70 | 0.88 | -0.37 |  | -0.13 | -0.13 |
| 47 | 0.89 | 0.36 | 0.37 | 0.94 | 0.88 | 1.36 | 0.15 | 0.60 | 0.70 | -0.43 |  | -0.30 | -0.30 |
| 48 |  |  |  | -0.94 | -0.92 | -1.61 | -0.15 | 0.06 | 0.06 | 0.37 |  | 0.41 | 0.44 |
| 49 | -0.14 | -0.45 | -0.49 | -0.77 | -0.87 | -1.32 | 0.52 | 0.14 | 0.14 |  |  |  |  |
| 50 | 0.09 | -0.49 | -0.54 | -0.77 | -0.88 | -1.38 |  |  |  | 0.77 |  | 0.51 | 0.56 |
| 51 | 0.54 | -0.42 | -0.45 | -0.66 | -0.66 | -0.79 | 0.33 | -0.20 | -0.20 | 0.31 |  | -0.02 | -0.02 |
| 52 | 0.09 | 0.09 | 0.09 | 0.83 | 0.74 | 0.96 | 0.09 | 0.05 | 0.05 | -0.14 |  | 0.26 | 0.27 |

| Sub5 | Sub5 | Sub5 | Sub6 | Sub6 | Sub6 | Sub7 | Sub7 | Sub7 | Sub8 | Sub8 | Sub8 |
| --- | --- | --- | --- | --- | --- | --- | --- | --- | --- | --- | --- |
| *r* | *sr* | *Z* | *r* | *sr* | *Z* | *r* | *sr* | *Z* | *r* | *sr* | *Z* |
| 0.43 | 0.57 | 0.65 |  |  |  | -0.14 | 0.63 | 0.73 | 0.93 | 0.17 | 0.17 |
| 0.26 | 0.18 | 0.19 |  |  |  | 0.14 | -0.12 | -0.12 | 0.75 | 0.00 | 0.00 |
| -0.09 | -0.16 | -0.16 | 0.26 | -0.03 | -0.03 | 0.89 | 0.21 | 0.21 | 0.93 | 0.17 | 0.17 |
| -0.09 | 0.13 | 0.13 | 0.77 | 0.52 | 0.58 | 0.14 | 0.45 | 0.49 | 0.72 | 0.76 | 1.00 |
| 0.14 | 0.09 | 0.09 | 0.71 | 0.77 | 1.02 | 0.54 | 0.37 | 0.39 | 0.32 | 0.08 | 0.08 |
| -0.09 | 0.13 | 0.13 | 0.26 | 0.32 | 0.33 | 0.60 | 0.81 | 1.14 | 0.38 | 0.25 | 0.26 |
| 0.03 | 0.44 | 0.47 | 0.14 | 0.29 | 0.30 | 0.03 | 0.15 | 0.15 | 0.70 | 0.34 | 0.35 |
| 0.49 | 0.44 | 0.47 | 0.77 | 0.35 | 0.36 | 0.14 | 0.67 | 0.81 | 0.81 | 0.51 | 0.56 |
| 0.49 | 0.44 | 0.47 | -0.09 | -0.11 | -0.11 | 0.43 | 0.36 | 0.37 | 0.93 | 0.17 | 0.17 |
| 0.43 | 0.57 | 0.65 | 0.77 | 0.35 | 0.36 | 0.37 | 0.92 | 1.61 | 0.90 | 0.42 | 0.45 |
| -0.03 | 0.64 | 0.76 | 0.31 | 0.24 | 0.25 | -0.26 | -0.40 | -0.42 | 0.84 | 0.68 | 0.82 |
| 0.49 | 0.44 | 0.47 | -0.26 | -0.67 | -0.80 | -0.43 | 0.22 | 0.22 | 0.93 | 0.17 | 0.17 |
| -0.14 | 0.34 | 0.35 | -0.37 | -0.87 | -1.32 | 0.37 | 0.27 | 0.28 | 0.93 | 0.17 | 0.17 |
| 0.14 | 0.09 | 0.09 | 0.60 | 0.22 | 0.23 | -0.03 | 0.50 | 0.55 | 0.75 | 0.51 | 0.56 |
| 0.31 | 0.05 | 0.05 | 0.26 | 0.75 | 0.98 | -0.71 | -0.33 | -0.34 | 0.12 | -0.08 | -0.08 |
| 0.20 | -0.04 | -0.04 | 0.49 | 0.72 | 0.90 | -0.03 | 0.43 | 0.46 | -0.06 | -0.59 | -0.68 |
| 0.20 | -0.04 | -0.04 | 0.26 | 0.49 | 0.54 | 0.03 | 0.65 | 0.78 | 0.06 | 0.17 | 0.17 |
| -0.20 | 0.33 | 0.34 | 0.94 | 0.65 | 0.77 | -0.20 | 0.76 | 1.00 | 0.75 | 0.51 | 0.56 |
| -0.26 | 0.68 | 0.83 | -0.09 | -0.28 | -0.29 | -0.03 | 0.50 | 0.55 | 0.55 | -0.25 | -0.26 |
| 0.49 | 0.44 | 0.47 | -0.31 | -0.77 | -1.01 | -0.14 | 0.48 | 0.52 | 0.90 | 0.42 | 0.45 |
| -0.31 | 0.24 | 0.24 | 0.49 | 0.54 | 0.61 | -0.14 | 0.63 | 0.73 | 0.72 | 0.76 | 1.00 |
| -0.09 | 0.20 | 0.21 | 0.37 | 0.43 | 0.46 | 0.09 | 0.16 | 0.16 | 0.99 | 0.34 | 0.35 |
| -0.09 | -0.16 | -0.16 | 0.20 | -0.39 | -0.41 | 0.31 | 0.70 | 0.86 | 0.90 | 0.08 | 0.08 |
| -0.43 | 0.08 | 0.08 | 0.60 | 0.31 | 0.32 | -0.60 | -0.17 | -0.17 | 0.84 | -0.08 | -0.08 |
| -0.09 | -0.16 | -0.16 | 0.66 | 0.58 | 0.67 | 0.09 | 0.81 | 1.11 | -0.03 | -0.08 | -0.08 |
| 0.03 | -0.21 | -0.21 | 0.60 | 0.57 | 0.65 | 0.49 | 0.94 | 1.74 | 0.23 | -0.59 | -0.68 |
| 0.03 | -0.21 | -0.21 | 0.49 | 0.72 | 0.90 | 0.03 | 0.87 | 1.33 | -0.06 | -0.59 | -0.68 |
| -0.31 | -0.12 | -0.12 | 0.37 | 0.78 | 1.04 | -0.31 | 0.45 | 0.49 | 0.52 | 0.68 | 0.82 |
| -0.14 | 0.34 | 0.35 | 0.37 | 0.34 | 0.36 | -0.60 | 0.41 | 0.43 | 0.99 | 0.34 | 0.35 |
| 0.71 | -0.10 | -0.10 | -0.26 | -0.67 | -0.80 | -0.54 | 0.20 | 0.21 | 0.75 | 0.76 | 1.00 |
| -0.26 | -0.69 | -0.85 | 0.09 | -0.76 | -1.00 | -0.31 | 0.53 | 0.59 | 0.93 | 0.59 | 0.68 |
| -0.54 | -0.30 | -0.31 | 0.03 | 0.01 | 0.01 | 0.03 | 0.08 | 0.08 | 0.58 | -0.17 | -0.17 |
| -0.37 | -0.49 | -0.54 | -0.60 | 0.13 | 0.13 | 0.20 | 0.32 | 0.33 | 0.99 | 0.34 | 0.35 |
| 0.03 | -0.43 | -0.46 | -0.37 | -0.26 | -0.26 | 0.37 | 0.71 | 0.88 | 0.52 | -0.17 | -0.17 |
| -0.09 | -0.59 | -0.68 | -0.09 | 0.42 | 0.44 | 0.77 | 0.48 | 0.52 | -0.06 | -0.59 | -0.68 |
| -0.54 | -0.30 | -0.31 | 0.83 | 0.62 | 0.73 | 0.03 | 0.65 | 0.78 | 0.20 | 0.17 | 0.17 |
| -0.60 | -0.02 | -0.02 | 0.43 | 0.62 | 0.72 | -0.20 | 0.62 | 0.72 | 0.23 | -0.59 | -0.68 |
| -0.37 | 0.37 | 0.39 | 0.60 | 0.74 | 0.96 | -0.43 | -0.07 | -0.07 | 0.38 | -0.17 | -0.17 |
| -0.20 | 0.33 | 0.34 | 0.71 | 0.16 | 0.16 | -0.31 | 0.45 | 0.49 | 0.81 | 0.68 | 0.82 |
| 0.09 | -0.13 | -0.13 | 0.31 | 0.07 | 0.07 | -0.77 | -0.19 | -0.20 | 0.64 | 0.42 | 0.45 |
| -0.43 | 0.00 | 0.00 | 0.54 | 0.47 | 0.51 | -0.77 | 0.17 | 0.17 | 0.90 | 0.42 | 0.45 |
| -0.77 | -0.05 | -0.05 | -0.03 | -0.53 | -0.59 | 0.14 | 0.17 | 0.17 | 0.55 | -0.08 | -0.08 |
| 0.37 | -0.30 | -0.31 | -0.71 | -0.42 | -0.45 | 0.37 | 0.71 | 0.88 | -0.17 | -0.34 | -0.35 |
| -0.14 | -0.53 | -0.59 | 0.66 | -0.11 | -0.11 | 0.20 | 0.32 | 0.33 | 0.55 | -0.51 | -0.56 |
| -0.66 | -0.32 | -0.33 | 0.31 | 0.51 | 0.56 | -0.03 | 0.43 | 0.46 | 0.52 | -0.17 | -0.17 |
| -0.43 | -0.14 | -0.14 | -0.09 | 0.42 | 0.44 | 0.49 | 0.51 | 0.56 | 0.32 | 0.51 | 0.56 |
| -0.43 | -0.14 | -0.14 | -0.37 | -0.43 | -0.46 | -0.09 | 0.78 | 1.04 | 0.46 | 0.51 | 0.56 |
| -0.49 | 0.14 | 0.14 | 0.03 | 0.27 | 0.27 | -0.09 | 0.42 | 0.45 | 0.29 | -0.08 | -0.08 |
| -0.49 | 0.14 | 0.14 | 0.89 | 0.72 | 0.91 | -0.66 | -0.10 | -0.10 | 0.09 | -0.59 | -0.68 |
| 0.09 | -0.13 | -0.13 | 0.71 | 0.16 | 0.16 | -0.77 | 0.17 | 0.17 | 0.84 | 0.25 | 0.26 |
| 0.03 | -0.43 | -0.46 | -0.37 | -0.87 | -1.32 | -0.26 | 0.46 | 0.50 | 0.72 | 0.34 | 0.35 |
| -0.66 | -0.32 | -0.33 | 0.77 | 0.52 | 0.58 | -0.37 | 0.59 | 0.68 | 0.90 | 0.08 | 0.08 |

| Sub9 | Sub9 | Sub9 | Sub10 | Sub10 | Sub10 | Sub11 | Sub11 | Sub11 | Sub12 | Sub12 | Sub12 |
| --- | --- | --- | --- | --- | --- | --- | --- | --- | --- | --- | --- |
| *r* | *sr* | *Z* | *r* | *sr* | *Z* | *r* | *sr* | *Z* | *r* | *sr* | *Z* |
| 0.09 | 0.01 | 0.01 | -0.20 | 0.34 | 0.36 | 0.71 | 0.58 | 0.67 | -0.83 | -0.30 | -0.31 |
| -0.09 | -0.19 | -0.20 | 0.09 | 0.59 | 0.67 | 0.54 | 0.34 | 0.35 | -0.43 | -0.56 | -0.63 |
| -0.26 | -0.25 | -0.26 | 0.31 | 0.39 | 0.41 | 0.77 | 0.31 | 0.33 | -0.49 | 0.24 | 0.24 |
| 0.09 | -0.17 | -0.18 | 0.49 | 0.59 | 0.68 | 0.71 | 0.35 | 0.37 | -0.49 | 0.24 | 0.24 |
| 0.03 | -0.02 | -0.02 | -0.37 | 0.14 | 0.14 | 0.37 | 0.09 | 0.09 | -0.37 | 0.10 | 0.10 |
| 0.03 | 0.05 | 0.05 | -0.43 | 0.12 | 0.12 | 0.77 | 0.31 | 0.33 | -0.54 | 0.07 | 0.07 |
| 0.09 | 0.49 | 0.54 | 0.54 | 0.75 | 0.97 | 0.66 | 0.03 | 0.04 | -0.60 | 0.06 | 0.06 |
| -0.09 | -0.16 | -0.16 | 0.37 | 0.41 | 0.44 | 0.37 | 0.03 | 0.04 | -0.89 | 0.01 | 0.01 |
| 0.60 | 0.66 | 0.79 | -0.43 | 0.12 | 0.12 | 0.49 | 0.31 | 0.33 | -0.71 | -0.45 | -0.48 |
| 0.77 | 0.72 | 0.90 | 0.43 | 0.85 | 1.24 | 0.66 | 0.15 | 0.15 | -0.77 | -0.29 | -0.30 |
| -0.26 | -0.21 | -0.22 | -0.14 | -0.33 | -0.34 | 0.60 | 0.59 | 0.69 | -0.26 | -0.37 | -0.39 |
| -0.09 | -0.19 | -0.20 | 0.09 | 0.45 | 0.48 | 0.60 | 0.30 | 0.31 | -0.60 | -0.75 | -0.97 |
| -0.26 | -0.21 | -0.22 | -0.09 | 0.25 | 0.25 | 0.77 | 0.31 | 0.33 | -0.54 | -0.74 | -0.95 |
| 0.03 | 0.38 | 0.41 | 0.66 | 0.65 | 0.78 | 0.66 | 0.03 | 0.04 | -0.60 | 0.22 | 0.22 |
| 0.03 | 0.38 | 0.41 | 0.26 | -0.05 | -0.05 | 0.49 | -0.09 | -0.09 | -0.43 | 0.25 | 0.26 |
| -0.14 | -0.08 | -0.08 | -0.09 | 0.25 | 0.25 | 0.37 | 0.09 | 0.09 | 0.14 | 0.83 | 1.20 |
| 0.03 | 0.38 | 0.41 | 0.37 | 0.55 | 0.62 | 0.09 | -0.61 | -0.70 | -0.60 | 0.06 | 0.06 |
| -0.26 | -0.25 | -0.26 | 0.77 | 0.14 | 0.14 | 0.26 | 0.05 | 0.05 | -0.43 | 0.25 | 0.26 |
| 0.54 | 0.22 | 0.23 | 0.03 | 0.70 | 0.87 | 0.66 | 0.15 | 0.15 | -0.94 | -0.49 | -0.53 |
| 0.49 | 0.71 | 0.88 | 0.83 | 0.57 | 0.65 | 0.37 | 0.03 | 0.04 | -0.77 | 0.03 | 0.03 |
| 0.26 | 0.25 | 0.26 | 0.03 | 0.15 | 0.15 | 0.09 | -0.20 | -0.20 | -0.94 | -0.32 | -0.34 |
| -0.14 | 0.03 | 0.03 | 0.20 | 0.63 | 0.73 | 0.09 | 0.44 | 0.48 | 0.71 | -0.04 | -0.04 |
| -0.14 | 0.03 | 0.03 | 0.60 | 0.91 | 1.52 | -0.26 | 0.07 | 0.07 | 0.60 | -0.06 | -0.06 |
| 0.14 | 0.34 | 0.35 | 0.26 | 0.37 | 0.39 | 0.83 | 0.28 | 0.29 | -0.31 | 0.27 | 0.28 |
| 0.20 | 0.55 | 0.62 | 0.83 | 0.57 | 0.65 | 0.66 | 0.03 | 0.04 | 0.26 | 0.53 | 0.59 |
| -0.14 | 0.29 | 0.30 | 0.71 | 0.12 | 0.12 | 0.60 | 0.07 | 0.07 | 0.03 | 0.49 | 0.54 |
| 0.03 | 0.24 | 0.24 | 0.66 | 0.37 | 0.39 | 0.71 | 0.35 | 0.37 | -0.26 | 0.60 | 0.70 |
| 0.03 | 0.05 | 0.05 | 0.54 | 0.33 | 0.35 | 0.60 | -0.10 | -0.11 | 0.14 | 0.83 | 1.20 |
| 0.54 | 0.22 | 0.23 | 0.83 | 0.57 | 0.65 | 0.66 | 0.15 | 0.15 | -0.09 | 0.63 | 0.74 |
| -0.09 | 0.10 | 0.10 | -0.03 | 0.41 | 0.43 | -0.03 | -0.19 | -0.19 | -0.49 | 0.24 | 0.24 |
| -0.49 | -0.15 | -0.15 | -0.31 | 0.03 | 0.03 | -0.03 | -0.19 | -0.19 | -0.09 | 0.47 | 0.51 |
| 0.03 | 0.09 | 0.09 | -0.60 | -0.49 | -0.54 | 0.14 | -0.12 | -0.12 | 0.09 | 0.50 | 0.55 |
| 0.14 | 0.34 | 0.35 | 0.14 | 0.47 | 0.51 | 0.09 | 0.33 | 0.34 | 0.37 | 0.23 | 0.23 |
| 0.26 | 0.21 | 0.22 | 0.31 | 0.39 | 0.41 | 0.77 | 0.49 | 0.54 | 0.09 | 0.18 | 0.18 |
| 0.54 | 0.81 | 1.13 | 0.49 | 0.73 | 0.93 | 0.09 | -0.26 | -0.26 | -0.89 | 0.01 | 0.01 |
| 0.09 | 0.19 | 0.20 | 0.71 | 0.40 | 0.42 | 0.71 | 0.35 | 0.37 | 0.03 | 0.33 | 0.34 |
| -0.09 | 0.29 | 0.29 | 0.03 | 0.56 | 0.64 | 0.71 | 0.35 | 0.37 | -0.54 | 0.07 | 0.07 |
| -0.37 | -0.02 | -0.02 | -0.26 | -0.37 | -0.39 | 0.60 | -0.10 | -0.11 | -0.09 | 0.63 | 0.74 |
| 0.49 | 0.41 | 0.44 | 0.20 | -0.34 | -0.36 | 0.71 | 0.00 | 0.00 | -0.26 | 0.60 | 0.70 |
| 0.77 | 0.35 | 0.36 | 0.14 | 0.33 | 0.34 | -0.26 | -0.75 | -0.97 | -0.77 | 0.19 | 0.19 |
| -0.43 | -0.01 | -0.01 | 0.43 | 0.57 | 0.65 | 0.14 | -0.35 | -0.37 | -0.09 | 0.47 | 0.51 |
| -0.54 | -0.55 | -0.62 | 0.94 | 0.62 | 0.72 | 0.09 | -0.02 | -0.02 | -0.49 | 0.24 | 0.24 |
| -0.26 | -0.21 | -0.22 | 0.26 | 0.37 | 0.39 | 0.09 | -0.26 | -0.26 | -0.20 | 0.29 | 0.30 |
|  |  |  | 0.31 | 0.39 | 0.41 | 0.77 | 0.31 | 0.33 | -0.26 | 0.44 | 0.47 |
| -0.09 | -0.19 | -0.20 | 0.71 | 0.40 | 0.42 | 0.60 | 0.54 | 0.60 | -0.43 | 0.25 | 0.26 |
|  |  |  | 0.14 | 0.33 | 0.34 | 0.37 | 0.09 | 0.09 | -0.31 | 0.27 | 0.28 |
| 0.26 | 0.25 | 0.26 | 0.14 | 0.33 | 0.34 | 0.43 | 0.41 | 0.43 | -0.66 | 0.21 | 0.21 |
| 0.71 | 0.72 | 0.91 | 0.14 | 0.33 | 0.34 | 0.60 | -0.10 | -0.11 | -0.26 | 0.28 | 0.29 |
| 0.14 | 0.52 | 0.58 | -0.31 | -0.25 | -0.26 | 0.60 | -0.10 | -0.11 | 0.03 | 0.65 | 0.78 |
| 0.37 | -0.09 | -0.09 | -0.03 | -0.15 | -0.15 | 0.77 | 0.31 | 0.33 | -0.83 | 0.18 | 0.18 |
| -0.09 | -0.01 | -0.01 | 0.09 | 0.17 | 0.17 | 0.20 | -0.15 | -0.15 | -0.77 | 0.19 | 0.19 |
| -0.31 | -0.39 | -0.41 | 0.49 | 0.31 | 0.32 | -0.20 | -0.66 | -0.80 | -0.49 | 0.08 | 0.08 |

| Sub13 | Sub13 | Sub13 | Sub14 | Sub14 | Sub14 | Sub15 | Sub15 | Sub15 | Sub16 | Sub16 | Sub16 |
| --- | --- | --- | --- | --- | --- | --- | --- | --- | --- | --- | --- |
| *r* | *sr* | *Z* | *r* | *sr* | *Z* | *r* | *sr* | *Z* | *r* | *sr* | *Z* |
| 0.71 | 0.28 | 0.29 | 0.03 | -0.22 | -0.23 | 0.00 | 0.16 | 0.16 | 0.32 | 0.67 | 0.81 |
| 0.71 | 0.28 | 0.29 | -0.20 | 0.09 | 0.09 | 0.49 | -0.08 | -0.08 | -0.03 | 0.58 | 0.66 |
| 0.20 | 0.49 | 0.53 | -0.14 | 0.21 | 0.21 | 0.90 | 0.32 | 0.33 | 0.93 | 0.53 | 0.60 |
| -0.03 | 0.21 | 0.21 | -0.14 | -0.58 | -0.67 | 0.35 | 0.22 | 0.23 | 0.20 | 0.52 | 0.58 |
| -0.03 | 0.54 | 0.60 | -0.14 | -0.81 | -1.12 | 0.14 | -0.46 | -0.50 | 0.41 | 0.40 | 0.42 |
| -0.14 | 0.73 | 0.92 | 0.26 | -0.53 | -0.59 | 0.64 | 0.27 | 0.28 | -0.61 | 0.31 | 0.32 |
| 0.54 | 0.57 | 0.65 | 0.26 | -0.53 | -0.59 | 0.75 | 0.29 | 0.30 | -0.17 | 0.60 | 0.69 |
| -0.09 | 0.31 | 0.32 | 0.54 | -0.15 | -0.15 | -0.06 | 0.15 | 0.15 | -0.14 | 0.43 | 0.46 |
| 0.77 | 0.84 | 1.24 | 0.31 | 0.04 | 0.04 | 0.70 | 0.28 | 0.29 | -0.12 | 0.50 | 0.54 |
| 0.49 | 0.66 | 0.80 | 0.43 | 0.28 | 0.29 | 0.35 | 0.38 | 0.40 | -0.64 | 0.13 | 0.13 |
| 0.49 | 0.34 | 0.35 | 0.43 | 0.51 | 0.56 | -0.03 | -0.17 | -0.17 | -0.09 | 0.56 | 0.64 |
| 0.03 | 0.44 | 0.48 | -0.77 | -0.33 | -0.34 | 0.26 | 0.21 | 0.21 | -0.17 | 0.60 | 0.69 |
| 0.14 | -0.07 | -0.07 | -0.66 | -0.09 | -0.09 | 0.35 | 0.38 | 0.40 | -0.17 | 0.60 | 0.69 |
| -0.09 | 0.63 | 0.75 | -0.14 | -0.81 | -1.12 | 0.70 | -0.20 | -0.20 | 0.55 | 0.26 | 0.27 |
| -0.09 | 0.63 | 0.75 | 0.03 | -0.67 | -0.82 | 0.55 | -0.23 | -0.23 | 0.41 | 0.16 | 0.17 |
| -0.31 | 0.03 | 0.03 | -0.14 | -0.81 | -1.12 | 0.41 | -0.25 | -0.26 | 0.29 | 0.13 | 0.13 |
| -0.37 | 0.45 | 0.49 | 0.26 | -0.53 | -0.59 | 0.87 | -0.01 | -0.01 | 0.12 | 0.21 | 0.21 |
| 0.09 | 0.35 | 0.36 | 0.43 | -0.39 | -0.41 | 0.67 | 0.44 | 0.47 | 0.03 | 0.59 | 0.68 |
| 0.77 | -0.14 | -0.14 | 0.14 | 0.13 | 0.13 | 0.29 | 0.21 | 0.22 | 0.46 | 0.76 | 1.01 |
| 0.60 | 0.15 | 0.15 | 0.71 | 0.32 | 0.33 | 0.70 | -0.04 | -0.04 | -0.43 | 0.47 | 0.51 |
| 0.37 | 0.53 | 0.59 | 0.14 | 0.13 | 0.13 | 0.32 | -0.27 | -0.27 | -0.70 | 0.23 | 0.23 |
| -0.77 | -0.19 | -0.19 | -0.14 | -0.02 | -0.02 | -0.03 | 0.32 | 0.33 | 0.00 | 0.53 | 0.59 |
| -0.77 | -0.19 | -0.19 | -0.09 | -0.24 | -0.24 | 0.99 | 0.33 | 0.35 | -0.23 | 0.58 | 0.67 |
| -0.09 | -0.35 | -0.36 | -0.14 | -0.81 | -1.12 | 0.14 | -0.46 | -0.50 | 0.32 | 0.32 | 0.33 |
| -0.77 | -0.19 | -0.19 | 0.14 | -0.66 | -0.79 | 0.41 | -0.25 | -0.26 | 0.20 | 0.05 | 0.05 |
| -0.31 | 0.03 | 0.03 | 0.03 | -0.67 | -0.82 | 0.23 | -0.28 | -0.29 | 0.41 | 0.16 | 0.17 |
| -0.20 | 0.17 | 0.17 | 0.09 | -0.55 | -0.62 | 0.41 | -0.25 | -0.26 | -0.70 | -0.47 | -0.52 |
| -0.60 | -0.15 | -0.15 | 0.49 | -0.50 | -0.55 | 0.41 | -0.25 | -0.26 | -0.12 | -0.09 | -0.09 |
| -0.60 | -0.47 | -0.52 | -0.31 | -0.61 | -0.70 | -0.23 | -0.20 | -0.20 | -0.35 | -0.15 | -0.15 |
| 0.26 | 0.39 | 0.41 | 0.49 | 0.29 | 0.30 | 0.03 | -0.32 | -0.33 | -0.70 | 0.11 | 0.11 |
| 0.14 | 0.58 | 0.66 | 0.31 | -0.52 | -0.58 | -0.03 | 0.32 | 0.33 | -0.70 | 0.23 | 0.23 |
| -0.54 | -0.24 | -0.25 | -0.14 | -0.58 | -0.67 |  |  |  | -0.20 | -0.64 | -0.76 |
| -0.54 | 0.08 | 0.08 | -0.31 | -0.49 | -0.54 | -0.32 | -0.70 | -0.87 | -0.64 | -0.58 | -0.66 |
| -0.49 | -0.34 | -0.35 | 0.14 | -0.66 | -0.79 | -0.12 | 0.63 | 0.73 | -0.20 | 0.06 | 0.06 |
| 0.37 | -0.13 | -0.13 | 0.03 | -0.67 | -0.82 | -0.12 | -0.67 | -0.80 | 0.35 | 0.50 | 0.55 |
| -0.31 | 0.03 | 0.03 | -0.14 | -0.70 | -0.86 | 0.23 | -0.28 | -0.29 | -0.06 | 0.39 | 0.42 |
| -0.43 | 0.55 | 0.62 | -0.20 | -0.93 | -1.65 | 0.41 | -0.09 | -0.09 | -0.75 | -0.25 | -0.26 |
| -0.43 | -0.43 | -0.46 | 0.43 | -0.39 | -0.41 | 0.64 | 0.27 | 0.28 | -0.20 | 0.53 | 0.59 |
| -0.66 | -0.05 | -0.05 | 0.43 | -0.51 | -0.56 | 0.29 | 0.37 | 0.39 | -0.49 | 0.34 | 0.35 |
| 0.31 | 0.62 | 0.73 | 0.43 | -0.51 | -0.56 | 0.81 | 0.46 | 0.50 | -0.64 | 0.13 | 0.13 |
| 0.26 | 0.72 | 0.90 | 0.66 | 0.09 | 0.09 | -0.61 | -0.75 | -0.98 | -0.75 | -0.25 | -0.26 |
| 0.14 | 0.91 | 1.51 | 0.14 | -0.66 | -0.79 | -0.23 | -0.36 | -0.38 | 0.06 | 0.31 | 0.32 |
| 0.26 | 0.39 | 0.41 | -0.26 | -0.49 | -0.53 | -0.17 | -0.84 | -1.21 | -0.20 | -0.64 | -0.76 |
| -0.26 | -0.06 | -0.06 | 0.43 | -0.51 | -0.56 | -0.06 | -0.49 | -0.54 | 0.35 | 0.50 | 0.55 |
| -0.60 | -0.15 | -0.15 | 0.03 | -0.67 | -0.82 | -0.26 | -0.37 | -0.39 | -0.32 | 0.50 | 0.55 |
| -0.66 | -0.05 | -0.05 | -0.31 | -0.61 | -0.70 | -0.46 | -0.24 | -0.25 | -0.29 | 0.22 | 0.22 |
| 0.37 | 0.53 | 0.59 | -0.26 | -0.49 | -0.53 | 0.41 | -0.09 | -0.09 | -0.12 | -0.32 | -0.33 |
| -0.77 | -0.19 | -0.19 | 0.14 | -0.66 | -0.79 | 0.58 | -0.06 | -0.06 | -0.64 | -0.46 | -0.50 |
|  |  |  | -0.14 | -0.70 | -0.86 | 0.29 | 0.37 | 0.39 | -0.49 | 0.46 | 0.49 |
| 0.31 | -0.36 | -0.38 | 0.26 | -0.42 | -0.44 | 0.81 | 0.46 | 0.50 | 0.09 | 0.02 | 0.02 |
| 0.49 | 0.34 | 0.35 | 0.66 | -0.25 | -0.25 | 0.93 | 0.32 | 0.33 | -0.75 | 0.21 | 0.22 |
| -0.31 | 0.36 | 0.38 | 0.66 | -0.25 | -0.25 | -0.17 | -0.19 | -0.19 | -0.06 | -0.07 | -0.07 |

| Sub17 | Sub17 | Sub17 | Sub18 | Sub18 | Sub18 | Sub19 | Sub19 | Sub19 | Sub20 | Sub20 | Sub20 |
| --- | --- | --- | --- | --- | --- | --- | --- | --- | --- | --- | --- |
| *r* | *sr* | *Z* | *r* | *sr* | *Z* | *r* | *sr* | *Z* | *r* | *sr* | *Z* |
| -0.09 | 0.37 | 0.39 | 0.23 | 0.36 | 0.38 | 0.31 | 0.05 | 0.05 | 0.03 | 0.12 | 0.12 |
| -0.26 | 0.24 | 0.25 | -0.09 | 0.15 | 0.15 | 0.49 | 0.15 | 0.15 | 0.26 | -0.26 | -0.27 |
| 0.66 | 0.62 | 0.72 | 0.03 | 0.65 | 0.78 | 0.14 | -0.34 | -0.35 | 0.49 | 0.34 | 0.35 |
| 0.77 | 0.72 | 0.91 | -0.29 | -0.21 | -0.22 | -0.60 | -0.24 | -0.24 | 0.09 | 0.68 | 0.82 |
| 0.83 | 0.57 | 0.65 | -0.99 | -0.33 | -0.35 | -0.43 | -0.64 | -0.76 | 0.14 | 0.58 | 0.66 |
| 0.94 | 0.85 | 1.26 | 0.12 | -0.30 | -0.31 | -0.60 | -0.81 | -1.14 | 0.49 | 0.34 | 0.35 |
| 1.00 | 0.70 | 0.87 | -0.14 | 0.62 | 0.73 | -0.94 | -0.44 | -0.47 | -0.14 | 0.07 | 0.07 |
| 0.37 | 0.50 | 0.55 | -0.43 | 0.57 | 0.65 | -0.49 | 0.14 | 0.14 | 0.31 | -0.36 | -0.38 |
| -0.26 | 0.24 | 0.25 | -0.38 | 0.10 | 0.10 | -0.71 | -0.54 | -0.61 | -0.03 | -0.77 | -1.02 |
|  |  |  | -0.41 | 0.09 | 0.09 | 0.37 | -0.37 | -0.39 | 0.03 | -0.87 | -1.31 |
| 0.31 | 0.71 | 0.89 | 0.06 | 0.49 | 0.54 | 0.54 | -0.27 | -0.28 | 0.49 | 0.34 | 0.35 |
| -0.03 | 0.22 | 0.23 | 0.23 | -0.44 | -0.48 | 0.03 | 0.65 | 0.78 | 0.14 | -0.07 | -0.07 |
| 0.31 | 0.54 | 0.60 | -0.52 | -0.41 | -0.44 | -0.66 | 0.33 | 0.34 | 0.03 | 0.12 | 0.12 |
| 0.60 | 0.59 | 0.69 | -0.14 | -0.51 | -0.56 | -0.77 | -0.27 | -0.27 | 0.14 | 0.91 | 1.51 |
| 0.49 | 0.31 | 0.33 | -0.72 | -0.61 | -0.71 | -0.37 | 0.01 | 0.01 | -0.60 | -0.47 | -0.52 |
| 0.77 | 0.72 | 0.91 | -0.49 | -0.41 | -0.43 | -0.20 | -0.90 | -1.45 | 0.49 | 0.34 | 0.35 |
| 0.94 | 0.73 | 0.94 | -0.64 | 0.37 | 0.39 | -0.83 | -0.63 | -0.75 | 0.14 | 0.58 | 0.66 |
| 0.89 | 0.71 | 0.89 | 0.12 | 0.83 | 1.18 | -0.94 | -0.51 | -0.56 | -0.09 | 0.31 | 0.32 |
| -0.43 | 0.12 | 0.12 |  |  |  | -0.09 | 0.20 | 0.21 | 0.49 | -0.32 | -0.33 |
| -0.03 | 0.45 | 0.49 | -0.23 | -0.20 | -0.20 | 0.37 | -0.52 | -0.57 | 0.49 | -0.32 | -0.33 |
| -0.03 | 0.45 | 0.49 | -0.29 | 0.76 | 0.99 | 0.37 | 0.49 | 0.54 | 0.26 | 0.06 | 0.06 |
| -0.03 | 0.45 | 0.49 | -0.64 | -0.92 | -1.58 | -0.37 | 0.30 | 0.31 | -0.43 | -0.11 | -0.11 |
| -0.31 | 0.22 | 0.23 | -0.84 | -0.63 | -0.74 | -0.77 | 0.24 | 0.24 | -0.03 | -0.77 | -1.02 |
| 0.49 | 0.31 | 0.33 | -0.12 | -0.18 | -0.18 | -0.83 | -0.35 | -0.36 | -0.14 | 0.07 | 0.07 |
| 0.49 | 0.31 | 0.33 | 0.23 | -0.12 | -0.12 | -0.60 | -0.24 | -0.24 | 0.14 | -0.07 | -0.07 |
| 0.54 | 0.16 | 0.16 | -0.58 | -0.91 | -1.52 | -0.66 | -0.68 | -0.83 | 0.49 | 0.01 | 0.01 |
| 0.94 | 0.56 | 0.63 | -0.52 | -0.74 | -0.94 | -0.37 | -0.27 | -0.28 | 0.37 | 0.53 | 0.59 |
| 0.49 | 0.31 | 0.33 | -0.41 | 0.25 | 0.26 | -0.09 | 0.20 | 0.21 | -0.49 | -0.34 | -0.35 |
| 0.20 | 0.37 | 0.39 |  |  |  | -0.94 | -0.51 | -0.56 | 0.09 | 0.02 | 0.02 |
| -0.09 | 0.43 | 0.46 |  |  |  | -0.60 | -0.45 | -0.49 | -0.26 | -0.72 | -0.90 |
| 0.14 | 0.41 | 0.43 | -0.64 | -0.60 | -0.69 | 0.43 | -0.58 | -0.66 | 0.37 | -0.13 | -0.13 |
| 0.54 | 0.34 | 0.35 | 0.29 | 0.21 | 0.22 | -0.60 | 0.12 | 0.12 | 0.26 | -0.26 | -0.27 |
| 0.03 | 0.13 | 0.13 | -0.64 | -0.92 | -1.58 | -0.26 | 0.46 | 0.50 | -0.71 | -0.61 | -0.71 |
| 0.26 | 0.51 | 0.57 | 0.32 | 0.38 | 0.40 | -0.09 | -0.16 | -0.16 | -0.09 | -0.02 | -0.02 |
| 0.43 | 0.17 | 0.18 | -0.06 | -0.17 | -0.17 | -0.09 | -0.37 | -0.39 | -0.09 | -0.68 | -0.82 |
| 0.43 | 0.17 | 0.18 | -0.64 | -0.27 | -0.28 | 0.26 | 0.11 | 0.11 | -0.03 | -0.44 | -0.48 |
| 0.49 | 0.02 | 0.02 | -0.72 | -0.61 | -0.71 | 0.66 | 0.54 | 0.60 | 0.49 | 0.34 | 0.35 |
| 0.43 | -0.12 | -0.12 | 0.09 | -0.31 | -0.32 | -0.14 | -0.02 | -0.02 | -0.03 | 0.54 | 0.60 |
| 0.60 | 0.30 | 0.31 | 0.06 | 0.49 | 0.54 | -0.26 | -0.62 | -0.72 | 0.37 | -0.13 | -0.13 |
| 0.20 | 0.37 | 0.39 |  |  |  | -0.43 | -0.21 | -0.21 | 0.60 | 0.15 | 0.15 |
| 0.54 | 0.63 | 0.74 | -0.41 | 0.25 | 0.26 | -0.43 | -0.86 | -1.29 | 0.09 | -0.31 | -0.32 |
| 0.49 | 0.66 | 0.80 | 0.75 | 0.29 | 0.30 | -0.03 | -0.80 | -1.09 | 0.49 | 0.66 | 0.80 |
| 0.71 | 0.17 | 0.18 | -0.64 | -0.92 | -1.58 | -0.49 | 0.28 | 0.29 | -0.60 | -0.15 | -0.15 |
| 0.37 | 0.33 | 0.34 | 0.43 | 0.24 | 0.24 | -0.37 | -0.56 | -0.64 | -0.14 | 0.07 | 0.07 |
| 0.60 | 0.30 | 0.31 | 0.70 | 0.28 | 0.29 | -0.60 | -0.89 | -1.41 | 0.03 | 0.12 | 0.12 |
| 0.09 | -0.37 | -0.39 | -0.84 | -0.63 | -0.74 | 0.20 | 0.25 | 0.25 | -0.03 | -0.77 | -1.02 |
| 0.60 | 0.01 | 0.01 | -0.84 | -0.47 | -0.51 | 0.43 | 0.57 | 0.65 | -0.03 | 0.21 | 0.21 |
| 0.37 | 0.03 | 0.04 | -0.03 | -0.49 | -0.54 | 0.26 | 0.26 | 0.26 | 0.49 | 0.66 | 0.80 |
| 0.60 | 0.01 | 0.01 | 0.23 | -0.12 | -0.12 | 0.14 | 0.09 | 0.09 |  |  |  |
| 0.60 | 0.59 | 0.69 | -0.20 | 0.29 | 0.30 | -0.14 | 0.05 | 0.05 |  |  |  |
| 0.66 | 0.44 | 0.48 | -0.72 | 0.20 | 0.20 | -0.60 | 0.12 | 0.12 | 0.43 | 0.43 | 0.46 |
| 0.77 | 0.72 | 0.91 | -0.29 | 0.76 | 0.99 | -0.09 | -0.59 | -0.68 | -0.09 | 0.63 | 0.75 |

| Sub21 | Sub21 | Sub21 | Sub22 | Sub22 | Sub22 | Sub23 | Sub23 | Sub23 | Sub24 | Sub24 | Sub24 |
| --- | --- | --- | --- | --- | --- | --- | --- | --- | --- | --- | --- |
| *r* | *sr* | *Z* | *r* | *sr* | *Z* | *r* | *sr* | *Z* | *r* | *sr* | *Z* |
| -0.49 | -0.07 | -0.07 | 0.71 | 0.12 | 0.12 | -0.37 | -0.49 | -0.53 | 0.14 | -0.40 | -0.42 |
| -0.49 | 0.05 | 0.05 | 0.43 | 0.20 | 0.21 | -0.37 | -0.10 | -0.10 | 0.31 | -0.14 | -0.14 |
| -0.66 | -0.48 | -0.52 | 0.31 | 0.23 | 0.23 | -0.37 | 0.29 | 0.30 | 0.37 | 0.09 | 0.09 |
| -0.43 | -0.05 | -0.05 | 0.66 | 0.77 | 1.02 | -0.54 | -0.68 | -0.83 | 0.60 | 0.69 | 0.85 |
| -0.71 | 0.07 | 0.07 | 0.89 | 0.82 | 1.17 | -0.71 | -0.10 | -0.10 | 0.49 | 0.66 | 0.80 |
| -0.26 | -0.28 | -0.29 | 0.14 | 0.70 | 0.86 | -0.43 | -0.29 | -0.30 | 0.60 | 0.69 | 0.85 |
| -0.26 | -0.28 | -0.29 | 0.37 | 0.80 | 1.11 | -0.83 | -0.49 | -0.53 | 0.43 | 0.32 | 0.34 |
| -0.49 | 0.05 | 0.05 | 0.66 | 0.82 | 1.16 | 0.20 | 0.68 | 0.83 | 0.31 | -0.47 | -0.51 |
| -0.60 | -0.26 | -0.27 | 0.66 | 0.41 | 0.44 | -0.09 | 0.49 | 0.53 | 0.03 | -0.43 | -0.46 |
| -0.71 | -0.26 | -0.26 | 0.94 | 0.63 | 0.75 |  |  |  | -0.09 | -0.46 | -0.49 |
| -0.49 | 0.05 | 0.05 | 0.60 | 0.14 | 0.14 | -0.03 | 0.68 | 0.83 | -0.26 | -0.06 | -0.06 |
| -0.66 | 0.16 | 0.17 | 0.31 | -0.18 | -0.18 | 0.03 | 0.49 | 0.53 | -0.14 | -0.47 | -0.51 |
| -0.89 | -0.47 | -0.51 | 0.31 | 0.23 | 0.23 | -0.26 | -0.10 | -0.10 | 0.37 | 0.09 | 0.09 |
| -0.43 | 0.15 | 0.15 | 0.60 | 0.45 | 0.48 | -0.77 | 0.10 | 0.10 | 0.60 | 0.69 | 0.85 |
| -0.14 | 0.00 | 0.00 | 0.60 | 0.45 | 0.48 | -0.77 | -0.29 | -0.30 | 0.89 | 0.76 | 1.00 |
| -0.71 | -0.46 | -0.49 | 0.37 | 0.34 | 0.36 | -0.83 | -0.49 | -0.53 | 0.37 | 0.09 | 0.09 |
| -0.71 | -0.46 | -0.49 | 0.26 | 0.32 | 0.33 | -0.09 | -0.29 | -0.30 | 0.60 | 0.26 | 0.26 |
| -0.83 | -0.65 | -0.78 | 0.60 | 0.91 | 1.52 | -0.31 | 0.49 | 0.53 | 0.49 | -0.21 | -0.21 |
| -0.49 | 0.05 | 0.05 | 0.66 | 0.41 | 0.44 | 0.71 | 0.88 | 1.37 | -0.26 | -0.50 | -0.55 |
| -0.20 | 0.22 | 0.23 | 0.71 | 0.12 | 0.12 | -0.26 | 0.68 | 0.83 | -0.37 | -0.53 | -0.59 |
| -0.66 | 0.00 | 0.00 | 0.31 | -0.18 | -0.18 |  |  |  | -0.37 | -0.53 | -0.59 |
| -0.54 | 0.00 | 0.00 | 0.26 | -0.04 | -0.04 | -0.03 | -0.10 | -0.10 | 0.31 | 0.08 | 0.08 |
| -0.77 | 0.05 | 0.05 | 0.77 | 0.34 | 0.35 | -0.49 | -0.10 | -0.10 | 0.37 | 0.09 | 0.09 |
| -0.94 | -0.49 | -0.53 | 0.54 | 0.23 | 0.24 | -0.71 | -0.10 | -0.10 | 0.09 | 0.35 | 0.36 |
| -0.71 | -0.46 | -0.49 | 0.49 | -0.29 | -0.30 | -0.60 | 0.29 | 0.30 | 0.89 | 0.33 | 0.34 |
| -0.60 | -0.46 | -0.50 | 0.14 | 0.70 | 0.86 | -0.94 | -0.49 | -0.53 | 0.89 | 0.76 | 1.00 |
| -0.77 | -0.64 | -0.75 | 0.26 | 0.32 | 0.33 | -0.60 | -0.49 | -0.53 | 0.77 | 0.30 | 0.31 |
| -0.89 | -0.63 | -0.74 | 0.20 | -0.26 | -0.26 | -0.66 | 0.10 | 0.10 | 0.77 | 0.30 | 0.31 |
| -0.83 | -0.49 | -0.54 | 0.77 | 0.39 | 0.41 | -0.43 | 0.49 | 0.53 | 0.03 | -0.87 | -1.31 |
| -0.20 | 0.22 | 0.23 | 0.71 | 0.58 | 0.66 | -0.26 | 0.68 | 0.83 | -0.26 | -0.50 | -0.55 |
| -0.77 | 0.05 | 0.05 | 0.66 | 0.36 | 0.38 | 0.09 | 0.68 | 0.83 | 0.03 | 0.01 | 0.01 |
| -0.77 | -0.60 | -0.69 | 0.60 | 0.91 | 1.52 | -0.26 | 0.29 | 0.30 | -0.09 | -0.02 | -0.02 |
| -0.71 | -0.18 | -0.18 | 0.77 | 0.80 | 1.09 | -0.03 | -0.10 | -0.10 | 0.14 | 0.25 | 0.26 |
| -0.43 | -0.69 | -0.85 | -0.03 | -0.31 | -0.32 | -0.49 | 0.29 | 0.30 | 0.94 | 0.56 | 0.63 |
| -0.94 | -0.49 | -0.53 | 0.37 | 0.04 | 0.04 | -0.83 | -0.10 | -0.10 | 0.89 | 0.33 | 0.34 |
| -0.54 | 0.04 | 0.04 | -0.03 | -0.31 | -0.32 | -0.83 | -0.49 | -0.53 | 0.77 | 0.74 | 0.94 |
| -0.71 | -0.74 | -0.95 | -0.09 | -0.12 | -0.12 | -0.89 | -0.68 | -0.83 | 0.94 | 0.56 | 0.63 |
| -0.77 | -0.64 | -0.75 | 0.49 | 0.42 | 0.45 | -0.89 | 0.10 | 0.10 | 0.77 | 0.30 | 0.31 |
| -0.94 | -0.49 | -0.53 | 0.26 | 0.73 | 0.92 | -0.60 | -0.10 | -0.10 | 0.71 | 0.50 | 0.55 |
| -0.83 | -0.49 | -0.54 | 0.43 | 0.20 | 0.21 | -0.26 | 0.29 | 0.30 | 0.03 | -0.43 | -0.46 |
| -0.83 | -0.69 | -0.85 | 0.43 | 0.20 | 0.21 | -0.26 | 0.68 | 0.83 | 0.14 | -0.29 | -0.30 |
| -0.94 | -0.73 | -0.92 | 0.43 | 0.20 | 0.21 | 0.49 | 0.88 | 1.37 | 0.26 | 0.94 | 1.70 |
| -0.83 | -0.49 | -0.54 | 0.71 | 0.94 | 1.71 | -0.83 | -0.10 | -0.10 | -0.09 | -0.35 | -0.36 |
| -0.31 | -0.46 | -0.49 | -0.03 | 0.45 | 0.49 | -0.31 | 0.49 | 0.53 | 0.03 | -0.21 | -0.21 |
| -0.09 | -0.31 | -0.32 | -0.37 | -0.34 | -0.36 | -0.49 | 0.29 | 0.30 | 0.43 | 0.87 | 1.33 |
| -0.49 | -0.67 | -0.81 | -0.20 | -0.61 | -0.71 | -0.60 | 0.29 | 0.30 | 0.77 | 0.30 | 0.31 |
| -0.71 | -0.18 | -0.18 | -0.49 | 0.29 | 0.30 | -1.00 | -0.29 | -0.30 | 0.89 | 0.33 | 0.34 |
| -0.26 | 0.00 | 0.00 | 0.43 | 0.61 | 0.71 | -0.83 | 0.29 | 0.30 | 0.31 | 0.40 | 0.43 |
| -0.83 | -0.49 | -0.54 | 0.26 | 0.73 | 0.92 | -0.94 | -0.10 | -0.10 | 0.60 | 0.80 | 1.10 |
| -0.37 | 0.01 | 0.01 | -0.77 | -0.85 | -1.25 | -0.77 | -0.29 | -0.30 | 0.77 | 0.41 | 0.43 |
| -0.94 | -0.49 | -0.53 | -0.26 | -0.27 | -0.27 | -0.09 | 0.10 | 0.10 | 0.37 | -0.02 | -0.02 |
| -0.71 | -0.74 | -0.95 | 0.14 | -0.43 | -0.45 | -0.37 | 0.29 | 0.30 | 0.37 | 0.42 | 0.45 |

| Sub25 | Sub25 | Sub25 | Sub26 | Sub26 | Sub26 | Sub27 | Sub27 | Sub27 |
| --- | --- | --- | --- | --- | --- | --- | --- | --- |
| *r* | *sr* | *Z* | *r* | *sr* | *Z* | *r* | *sr* | *Z* |
| -0.77 | -0.26 | -0.27 | 0.49 | -0.08 | -0.08 | 0.31 | 0.82 | 1.16 |
| 0.03 | 0.17 | 0.17 | -0.31 | -0.54 | -0.60 | 0.37 | 0.68 | 0.82 |
| -0.49 | -0.26 | -0.27 | -0.71 | -0.45 | -0.48 | 0.20 | 0.79 | 1.06 |
| -0.43 | -0.08 | -0.08 | -0.14 | -0.83 | -1.20 | 0.09 | 0.71 | 0.89 |
| -0.83 | -0.33 | -0.34 | -0.31 | -0.70 | -0.87 | -0.09 | 0.30 | 0.31 |
| -0.37 | 0.20 | 0.21 | -0.31 | -0.86 | -1.30 | -0.26 | 0.33 | 0.34 |
| -0.77 | -0.04 | -0.04 | -0.09 | -0.50 | -0.55 | -0.71 | 0.03 | 0.03 |
| 0.54 | 0.88 | 1.37 | -0.26 | 0.28 | 0.29 | 0.31 | 0.82 | 1.16 |
| 0.20 | 0.70 | 0.87 | 0.26 | -0.28 | -0.29 | 0.26 | 0.60 | 0.70 |
| 0.26 | 0.49 | 0.54 | 0.66 | 0.27 | 0.28 | -0.03 | 0.64 | 0.75 |
| -0.66 | -0.13 | -0.13 | -0.37 | -0.23 | -0.23 | 0.43 | 0.89 | 1.45 |
| -0.77 | -0.04 | -0.04 | -0.31 | -0.22 | -0.22 | 0.37 | 0.68 | 0.82 |
| -0.43 | -0.58 | -0.66 | -0.37 | -0.39 | -0.41 | 0.14 | 0.57 | 0.64 |
| -0.43 | -0.75 | -0.96 | -0.49 | -0.73 | -0.93 | 0.60 | 0.95 | 1.80 |
| -0.31 | 0.60 | 0.70 | -0.31 | -0.70 | -0.87 | -0.14 | 0.28 | 0.29 |
| -0.43 | -0.08 | -0.08 | -0.14 | -0.83 | -1.20 | -0.03 | 0.43 | 0.47 |
| -0.54 | 0.23 | 0.23 | -0.14 | -0.83 | -1.20 | -0.43 | 0.15 | 0.15 |
| -0.49 | 0.13 | 0.13 | -0.20 | -0.36 | -0.37 | -0.26 | 0.24 | 0.25 |
| 0.37 | 0.74 | 0.94 | -0.31 | 0.11 | 0.11 | 0.60 | 0.95 | 1.80 |
| -0.09 | 0.37 | 0.39 | 0.60 | -0.06 | -0.06 | 0.03 | 0.69 | 0.85 |
| 0.09 | 0.35 | 0.36 | -0.09 | -0.34 | -0.35 | -0.26 | 0.49 | 0.53 |
| -0.94 | -0.35 | -0.36 | -0.49 | -0.41 | -0.43 | 0.43 | 0.89 | 1.45 |
| -0.94 | -0.35 | -0.36 | 0.37 | 0.71 | 0.89 | 0.26 | 0.80 | 1.11 |
| -0.43 | -0.75 | -0.96 | -0.43 | -0.56 | -0.63 | 0.20 | 0.66 | 0.80 |
| -0.60 | -0.45 | -0.48 | -0.31 | -0.70 | -0.87 | 0.37 | 0.56 | 0.63 |
| -0.83 | -0.33 | -0.34 | -0.31 | -0.70 | -0.87 | 0.20 | 0.66 | 0.80 |
| -0.71 | 0.14 | 0.14 | -0.14 | -0.51 | -0.56 | -0.14 | 0.32 | 0.33 |
| 0.09 | -0.09 | -0.09 | -0.14 | -0.83 | -1.20 | -0.03 | 0.39 | 0.42 |
| -0.60 | -0.45 | -0.48 | -0.09 | 0.15 | 0.15 | 0.77 | 0.84 | 1.21 |
| 0.03 | 0.45 | 0.48 | 0.31 | 0.70 | 0.87 | 0.20 | 0.79 | 1.06 |
| -0.26 | -0.05 | -0.05 | 0.20 | 0.52 | 0.58 | 0.37 | 0.68 | 0.82 |
| -0.49 | -0.48 | -0.52 | 0.14 | -0.62 | -0.73 | 0.26 | 0.48 | 0.52 |
| -0.66 | -0.57 | -0.65 | -0.37 | -0.23 | -0.23 | 0.37 | 0.52 | 0.57 |
| -1.00 | -0.47 | -0.51 | -0.37 | -0.23 | -0.23 | -0.14 | 0.28 | 0.29 |
| -0.71 | -0.69 | -0.85 | -0.20 | -0.36 | -0.37 | -0.31 | 0.19 | 0.19 |
| -0.49 | 0.13 | 0.13 | -0.31 | -0.70 | -0.87 | 0.31 | 0.46 | 0.49 |
| -0.31 | 0.38 | 0.40 | -0.14 | -0.51 | -0.56 | -0.09 | 0.42 | 0.44 |
| -0.49 | 0.13 | 0.13 | -0.09 | -0.66 | -0.80 | 0.09 | -0.18 | -0.18 |
| -0.37 | 0.26 | 0.27 | -0.31 | -0.54 | -0.60 | -0.09 | 0.30 | 0.31 |
| -0.77 | -0.04 | -0.04 | 0.03 | 0.33 | 0.34 | 0.31 | 0.82 | 1.16 |
| -0.09 | 0.37 | 0.39 | 0.77 | 0.46 | 0.49 | 0.49 | 0.91 | 1.54 |
| -0.14 | 0.53 | 0.58 | 0.26 | -0.28 | -0.29 | 0.09 | 0.39 | 0.41 |
| -0.26 | -0.44 | -0.47 | -0.14 | -0.51 | -0.56 | -0.09 | -0.23 | -0.23 |
| -0.83 | -0.55 | -0.61 | 0.26 | 0.53 | 0.59 | -0.26 | 0.24 | 0.25 |
| -0.09 | 0.65 | 0.77 | -0.54 | -0.42 | -0.44 | -0.60 | 0.06 | 0.06 |
| 0.49 | 0.76 | 0.99 | -0.31 | -0.54 | -0.60 | -0.77 | -0.03 | -0.03 |
| 0.14 | 0.19 | 0.20 | -0.60 | -0.26 | -0.27 | -0.54 | -0.16 | -0.17 |
| -0.14 | 0.25 | 0.25 | -0.49 | -0.41 | -0.43 | -0.26 | 0.24 | 0.25 |
| -0.60 | -0.01 | -0.01 | 0.09 | -0.47 | -0.51 | -0.26 | 0.24 | 0.25 |
| -0.60 | -0.01 | -0.01 | -0.14 | -0.51 | -0.56 | -0.37 | -0.07 | -0.07 |
| -0.37 | 0.15 | 0.15 | 0.14 | -0.62 | -0.73 | -0.03 | 0.39 | 0.42 |
| -0.09 | 0.65 | 0.77 | 0.26 | -0.28 | -0.29 | -0.26 | -0.04 | -0.04 |

Results from the Spearman correlation analysis (*r*), semi-partial correlation analysis (*sr*), and the Z scores (*Z*) derived from the Fisher’s *r*-to-*z* transformation for each of the 52 channels across all participants (Subjects 1~27).

**Table S2 Results of one sample *t*-test of mean Z obtained from** $\boldsymbol{\rho}_{\boldsymbol{Incongruency score.}\boldsymbol{\beta}_{\boldsymbol{Apply}}\boldsymbol{|WTP}}$ **and Mean R for each channel**

| Channel | Mean R | Mean Z | SD | *d* | *t* | *p* | 1-*β* | *df* |
| --- | --- | --- | --- | --- | --- | --- | --- | --- |
| 1 | 0.19 | 0.19 | 0.48 | 0.40 | 2.05 | 0.051 | 0.50 | 25 |
| 2 | 0.02 | 0.02 | 0.40 | 0.06 | 0.30 | 0.770 | 0.06 | 25 |
| 3 | 0.16 | 0.16 | 0.42 | 0.38 | 1.98 | 0.058 | 0.48 | 26 |
| **4** | **0.29** | **0.30** | **0.70** | **0.42** | **2.19** | **0.038** | 0.56 | 26 |
| 5 | 0.13 | 0.14 | 0.54 | 0.25 | 1.30 | 0.206 | 0.24 | 26 |
| 6 | 0.21 | 0.21 | 0.68 | 0.31 | 1.59 | 0.123 | 0.34 | 26 |
| **7** | **0.23** | **0.24** | **0.50** | **0.47** | **2.46** | **0.021** | 0.66 | 26 |
| **8** | **0.30** | **0.31** | **0.51** | **0.61** | **3.15** | **0.004** | 0.86 | 26 |
| 9 | 0.08 | 0.08 | 0.60 | 0.13 | 0.69 | 0.499 | 0.10 | 26 |
| 10 | 0.21 | 0.21 | 0.69 | 0.30 | 1.52 | 0.143 | 0.31 | 24 |
| **11** | **0.23** | **0.24** | **0.52** | **0.45** | **2.36** | **0.026** | 0.62 | 26 |
| 12 | 0.07 | 0.07 | 0.47 | 0.15 | 0.77 | 0.449 | 0.11 | 26 |
| 13 | -0.04 | -0.04 | 0.51 | -0.07 | -0.36 | 0.719 | 0.06 | 26 |
| **14** | **0.30** | **0.31** | **0.71** | **0.44** | **2.27** | **0.032** | 0.59 | 26 |
| 15 | 0.07 | 0.07 | 0.51 | 0.14 | 0.72 | 0.477 | 0.11 | 26 |
| 16 | -0.01 | -0.01 | 0.64 | -0.01 | -0.04 | 0.965 | 0.05 | 26 |
| 17 | 0.14 | 0.15 | 0.54 | 0.27 | 1.41 | 0.170 | 0.27 | 26 |
| **18** | **0.32** | **0.33** | **0.59** | **0.55** | **2.88** | **0.008** | 0.79 | 26 |
| **19** | **0.27** | **0.28** | **0.65** | **0.43** | **2.18** | **0.039** | 0.55 | 25 |
| 20 | 0.16 | 0.16 | 0.51 | 0.31 | 1.64 | 0.114 | 0.35 | 26 |
| **21** | **0.20** | **0.20** | **0.40** | **0.50** | **2.54** | **0.018** | 0.69 | 25 |
| 22 | 0.15 | 0.16 | 0.51 | 0.30 | 1.58 | 0.127 | 0.33 | 26 |
| 23 | 0.19 | 0.19 | 0.58 | 0.32 | 1.69 | 0.104 | 0.37 | 26 |
| 24 | -0.05 | -0.05 | 0.44 | -0.11 | -0.59 | 0.560 | 0.09 | 26 |
| 25 | 0.07 | 0.07 | 0.47 | 0.15 | 0.79 | 0.438 | 0.12 | 26 |
| 26 | 0.01 | 0.01 | 0.73 | 0.02 | 0.09 | 0.930 | 0.05 | 26 |
| 27 | 0.08 | 0.08 | 0.59 | 0.13 | 0.69 | 0.494 | 0.10 | 26 |
| 28 | 0.13 | 0.13 | 0.57 | 0.23 | 1.19 | 0.246 | 0.21 | 26 |
| 29 | 0.08 | 0.08 | 0.54 | 0.16 | 0.80 | 0.430 | 0.12 | 25 |
| 30 | 0.17 | 0.17 | 0.52 | 0.33 | 1.67 | 0.107 | 0.36 | 25 |
| 31 | 0.10 | 0.10 | 0.54 | 0.19 | 0.99 | 0.330 | 0.16 | 25 |
| 32 | -0.08 | -0.09 | 0.52 | -0.16 | -0.83 | 0.413 | 0.13 | 25 |
| 33 | -0.09 | -0.09 | 0.61 | -0.14 | -0.73 | 0.471 | 0.11 | 26 |
| 34 | 0.00 | 0.00 | 0.48 | 0.00 | -0.02 | 0.985 | 0.05 | 26 |
| 35 | -0.08 | -0.08 | 0.57 | -0.13 | -0.69 | 0.497 | 0.10 | 26 |
| 36 | 0.10 | 0.10 | 0.48 | 0.21 | 1.10 | 0.281 | 0.19 | 26 |
| 37 | 0.01 | 0.01 | 0.62 | 0.01 | 0.06 | 0.955 | 0.05 | 26 |
| 38 | 0.08 | 0.08 | 0.46 | 0.18 | 0.96 | 0.347 | 0.15 | 26 |
| 39 | 0.19 | 0.19 | 0.47 | 0.41 | 2.12 | 0.044 | 0.53 | 26 |
| 40 | 0.14 | 0.14 | 0.44 | 0.31 | 1.58 | 0.126 | 0.33 | 25 |
| 41 | 0.21 | 0.22 | 0.67 | 0.32 | 1.68 | 0.105 | 0.37 | 26 |
| 42 | 0.25 | 0.25 | 0.76 | 0.33 | 1.72 | 0.098 | 0.38 | 26 |
| 43 | -0.19 | -0.19 | 0.65 | -0.29 | -1.52 | 0.141 | 0.31 | 26 |
| 44 | -0.02 | -0.02 | 0.48 | -0.03 | -0.17 | 0.864 | 0.05 | 25 |
| 45 | 0.05 | 0.05 | 0.57 | 0.09 | 0.46 | 0.652 | 0.07 | 26 |
| 46 | 0.03 | 0.03 | 0.56 | 0.06 | 0.32 | 0.753 | 0.06 | 25 |
| 47 | 0.16 | 0.16 | 0.48 | 0.33 | 1.74 | 0.094 | 0.39 | 26 |
| 48 | 0.06 | 0.06 | 0.53 | 0.12 | 0.61 | 0.549 | 0.09 | 25 |
| 49 | 0.03 | 0.03 | 0.60 | 0.05 | 0.25 | 0.806 | 0.06 | 23 |
| 50 | -0.07 | -0.07 | 0.50 | -0.13 | -0.66 | 0.518 | 0.10 | 24 |
| 51 | -0.04 | -0.04 | 0.45 | -0.09 | -0.49 | 0.626 | 0.08 | 26 |
| 52 | 0.12 | 0.12 | 0.54 | 0.22 | 1.13 | 0.269 | 0.19 | 26 |

Results from the one-sample *t*-test differed from 0 for the mean *Z* derived from $\rho_{Incongruency score.\beta_{Apply}|WTP}$ for 52 channels with the following values: *t*: *t*-values; SD: standard deviation; *d*: Cohen’s *d; p*: *p*-values; *1-β*: power, n=27. Values with *p*<0.05 are in bold and as channel 8 only has power 1-*β*>0.8, those data are in red. Additionally, the Mean R, calculated from the Mean Z using the inverse Fisher’s *z*-to-*r* transformation, is also provided.
